# The Mammalian Membrane Microenvironment Regulates the Sequential Attachment of Bacteria to Host Cells

**DOI:** 10.1128/mBio.01392-21

**Published:** 2021-08-03

**Authors:** Xavier Pierrat, Jeremy P. H. Wong, Zainebe Al-Mayyah, Alexandre Persat

**Affiliations:** a Institute of Bioengineering and Global Health Institute, School of Life Sciences, Ecole Polytechnique Fédérale de Lausanne, Lausanne, Switzerland; University of Washington

**Keywords:** adhesins, autotransporter proteins, cell adhesion, cell membranes, cytoskeleton, microfluidics, glycocalyx, membrane biophysics

## Abstract

Pathogen attachment to host tissue is critical in the progress of many infections. Bacteria use adhesion *in vivo* to stabilize colonization and subsequently regulate the deployment of contact-dependent virulence traits. To specifically target host cells, they decorate themselves with adhesins, proteins that bind to mammalian cell surface receptors. One common assumption is that adhesin-receptor interactions entirely govern bacterial attachment. However, how adhesins engage with their receptors in an *in vivo*-like context remains unclear, in particular under the influence of a heterogeneous mechanical microenvironment. We here investigate the biophysical processes governing bacterial adhesion to host cells using a tunable adhesin-receptor system. By dynamically visualizing attachment, we found that bacterial adhesion to host cell surface, unlike adhesion to inert surfaces, involves two consecutive steps. Bacteria initially attach to their host without engaging adhesins. This step lasts about 1 min, during which bacteria can easily detach. We found that at this stage, the glycocalyx, a layer of glycosylated proteins and lipids, shields the host cell by keeping adhesins away from their receptor ligand. In a second step, adhesins engage with their target receptors to strengthen attachment for minutes to hours. The active properties of the membrane, endowed by the actin cytoskeleton, strengthen specific adhesion. Altogether, our results demonstrate that adhesin-ligand binding is not the sole regulator of bacterial adhesion. In fact, the host cell’s surface mechanical microenvironment mediates the physical interactions between host and bacteria, thereby playing an essential role in the onset of infection.

## INTRODUCTION

In the wild, bacteria predominantly live associated with surfaces. Their sessile lifestyle confers fitness advantages such as protection from predators and improved access to nutrients (1). In the context of host colonization, the transition between planktonic and sessile lifestyles plays a functional role in mediating host-microbe interactions. Indeed, attachment to host tissue, more specifically to cells, is often a critical first step toward infection or commensalism (2, 3). As a result, the dynamics of attachment of single bacteria to host cells can dramatically influence the outcome of infection or regulate host-microbiota homeostasis (4).

Bacterial adhesion to abiotic materials greatly contributes to biofouling and contamination of indwelling medical devices. Multiple physicochemical properties of the surface mediate adhesion to inert materials, including charge, hydrophobicity, and conditioning (5). In addition, mechanical properties of the material such as stiffness and surrounding fluid flow regulate attachment strength and dynamics (6–8). The understanding of adhesion to abiotic materials provides us with only rudimentary insights on adhesion to biological tissue. More specifically, the physical and biological complexity of biotic surfaces remains overlooked when making the analogy between living and inert materials. The surface of host mammalian cells is composed of a soft lipid bilayer densely packed with surface proteins (9). In addition, it is a dynamic surface, permanently rearranging itself under the action of forces such as the ones generated by the cytoskeleton. Finally, in contrast with abiotic adhesion, bacterial attachment to host cell involves specific molecular interactions (3). As a result, drawing analogies between biotic and abiotic adhesion can be informative but may overlook critical physical and biological regulators.

Pathogens and commensals alike express proteins at their surfaces that specifically bind to host membrane receptors. These cell-type-specific adhesins promote tissue tropism during infection or colonization (10). These can be classified in categories that reflect their structure and molecular mechanism of display. Adhesins from the autotransporter family are exposed immediately near the bacterial cell envelope (11). Their structure includes an outer membrane beta-barrel scaffold and an inner alpha helix that holds a passenger domain. This domain often includes its ligand-binding domain (12). Intimin is an autotransporter adhesin from enteropathogenic and enterohemorrhagic Escherichia coli that mediates attachment to gut epithelial cells. Intimin binds to Tir receptors at the host membrane that have been preemptively translocated by the bacterium (13, 14). Yersinia pseudotuberculosis uses invasin, which binds to beta integrins present at the host cell membrane, to initiate host cell entry during infection (15, 16). Similarly, Neisseria meningitidis uses NadA to invade host cells (17).

How the microenvironment of the host cell surface mediates the interaction between adhesins and their receptors remains unclear. Absolute bacterial count suggests that the membrane fluidity of host cells slightly decreases bacterial adhesion (18). At the molecular level of single adhesins, force spectroscopy measurements have helped characterize bond mechanics both on abiotic materials and on live cells (19). These have helped precisely identify exotic adhesin behavior such as the formation of catch bonds, which strengthen under an applied tensile force. The fimbria tip adhesin FimH notoriously forms a catch bond, allowing uropathogenic E. coli to strengthen adhesion in the urinary tract under flow (20–22). Studies of bacterial adhesion, including catch bonds, have mainly focused on detachment of bacteria, where adhesive force balances externally applied mechanical load (23). How the physical environment regulates bacterial approach and attachment to mammalian cell surfaces has yet to be systematically investigated in context.

The structure and biochemistry of many adhesin-receptor interactions have been well characterized (2, 3, 24). Several studies showed a direct correlation between the molecular adhesin-receptor kinetics and attachment behavior of single bacteria to their target host cell (5, 6, 22). In some pathogens, bacteria sequentially deploy multiple adhesins, thereby establishing a multistep process. For example, Salmonella first reversibly attaches the Fim adhesin and then irreversibly attaches using the type III secretion system (25). This two-step process involves active deployment of adhesins that also have an impact on host physiology. While the molecular mechanisms of adhesion are clear for specific adhesins, these do not illuminate the general biophysical rules of adhesions to host cells. In particular, it remains complex to decouple adhesive from toxic effects when investigating pathogen adhesion.

To investigate the intrinsic contributions of mechanics in the early steps of bacterial adhesion to host cells, we combined synthetic and biophysical approaches. We fine-tuned adhesion by engineering autotransporters for heterologous inducible display of a synthetic adhesin on a nonpathogenic strain of E. coli, targeting an inducible synthetic mammalian cell surface receptor (26). We found that the specific attachment of bacteria to host cells occurs in two consecutive steps. A first step is nonspecific, taking place within the first few seconds following contact. This is followed by the onset of specific adhesion resulting in nearly irreversible attachment on a longer timescale. We found that mechanobiological factors of the host cell surface, including membrane mechanics, flow, and glycocalyx, regulate each of the adhesion steps. Overall, we show that the biomechanical microenvironment of host tissues strongly regulates the adhesion behavior of bacteria to their target cells, indicating that this process cannot be solely reduced to adhesin-receptor interactions.

## RESULTS

### Synthetic adhesion to characterize bacterial attachment to host cells.

**FIG 1:**
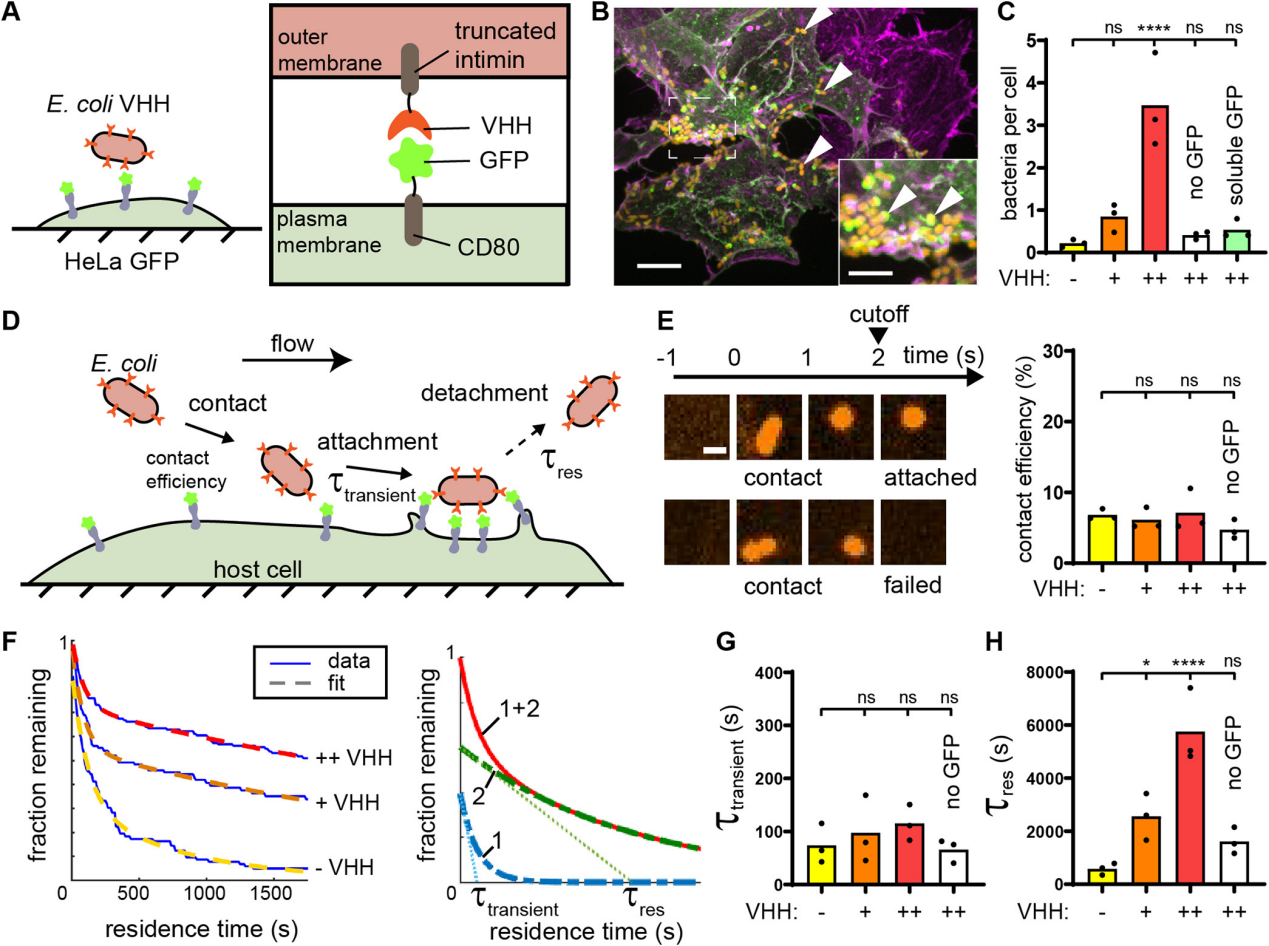
A synthetic adhesin-receptor system reveals a two-step mechanism of bacterial attachment to host cells. (A) Schematic of the synthetic adhesin-receptor system. E. coli cells display nanobody targeting GFP (VHH) fused to a truncated intimin autotransporter scaffold. HeLa cells display GFP receptors by fusion with the membrane-anchored CD80 scaffold (HeLa GFP). (B) In a mixed population of GFP^+^ (green) and GFP^−^ (purple) HeLa cells, E. coli (orange, indicated with white arrowheads) specifically binds to GFP^+^ cells. Actin stained with phalloidin (purple). Bars, 10 μm (main) and 5 μm (inset). (C) Bacterial count per HeLa cell increases with E. coli nanobody density. E. coli expressing VHH at low density or expressing VHH at high density but preincubated with soluble GFP only rarely binds to HeLa cells displaying GFP (“−,” “+,” and “++” correspond to no, low, and high VHH induction, respectively). (D) Dynamic visualizations of bacterial adhesion to HeLa cells under flow allow us to simultaneously monitor attachment and detachment events at multiple timescales. (E) Bacterial contact efficiency is independent of VHH density and GFP display. High-speed confocal imaging at 1 frame per second highlights bacterial populations that detach rapidly after contact. We considered bacteria attached if they stayed on the HeLa cell surface for more than 2 s. Bar, 2 μm. (F) We constructed residence time distributions using long-timescale tracking of attached bacteria (1 h). Bacteria adhering during the first 30 min were followed for 30 supplementary min in order to avoid artificial cropping of the data (see Materials and Methods). Bare E. coli and E. coli displaying low and high VHH levels have largely different residence time distributions. We fit these distributions using the sum of two exponentials to highlight two characteristic timescales, τ_transient_ and τ_res_ (right illustrative graph). The single exponentials are shown in dashed green and blue, and their sum is the continuous red line. (G) The model parameter τ_transient_ is independent of the adhesin displayed. (H) In contrast, the characteristic residence time τ_res_ increases with nanobody density. Statistical tests: one-way analysis of variance (ANOVA) followed by Dunnett’s *post hoc* test (****, *P* < 10^−4^; *, *P* < 0.05; ns, not significant).

To systematically probe bacterial adhesion to host cells without relying on virulence factors, we engineered an exogenous adhesin in a nonflagellated E. coli and cognate receptor in HeLa cells (Fig. 1A). As adhesin, we display a tetracycline-inducible anti-green fluorescent protein (anti-GFP) nanobody (camelid single-domain variable heavy chain [VHH]) using a truncated intimin scaffold (26, 27). The N-terminal domain consists in a beta-barrel associated with the bacterial outer membrane, through which spans an alpha helix displaying the synthetic passenger domain (see Fig. S1A in the supplemental material). Two out of four immunoglobulin-like structures and the lectin-like domain of the passenger domain of wild-type intimin are replaced with a hemagglutinin (HA) tag and VHH domain (26, 28). By staining with recombinant GFP and quantifying the fluorescence signal at the surface of single bacteria induced with increasing tetracycline concentrations, we generated titration curves allowing us to fine-tune the density of displayed VHH (Fig. S1D and E). To display receptor GFP ligand for the synthetic adhesin at the surface of HeLa cells, we displayed a doxycycline-inducible GFP fusion to a CD80 receptor anchored in the plasma membrane (Fig. S1B) (29). Direct visualization of the fluorescence signal localized at the cell plasma membrane can confirm and help quantify receptor density (Fig. S1F).

10.1128/mBio.01392-21.2 FIG S1 Synthetic VHH adhesins and GFP receptor constructs. (A) Schematic of the VHH display genetic construct. E. coli displays anti-GFP nanobodies (VHH) based on a tetracycline-inducible promoter (tet) and a truncated intimin that consists in a signal peptide (SP), a beta-barrel anchored in the outer membrane, and an alpha helix crossing the beta-barrel. The scaffold allows the display of an HA epitope tag (HA) and a nanobody anti-GFP (VHH). (B) Schematic of the GFP display construct. GFP was displayed at the surface of mammalian cells by means of a doxycycline-inducible promoter and a mammalian signal peptide (SP) and was anchored in the plasma membrane with a CD80 transmembrane domain (TM) and its C-terminal cytosolic domain. (C) Schematic of the GFP display genetic construct devoid of cytosolic component. GFP was displayed at the surface of mammalian cells by means of a constitutive cytomegalovirus (CMV) promoter and an insulin signal peptide (SP) and was anchored in the plasma membrane with a CD55 glycosylphosphatidylinositol anchor (GPI). (D) Widefield epifluorescence image of E. coli VHH stained with recombinant GFP. Bar, 2 μm. (E) Tetracycline titration followed by staining with recombinant GFP. Error bars represent standard deviation from triplicate fields of view. The solid line represents a Hill function fit. (F) Doxycycline titration on HeLa cells stably engineered with a doxycycline-inducible GFP display. Error bars represent standard deviation from triplicate fields. The solid line represents a Hill function fit. Download FIG S1, TIF file, 1.2 MB. Copyright © 2021 Pierrat et al. 2021 Pierrat et al. https://creativecommons.org/licenses/by/4.0/ This content is distributed under the terms of the Creative Commons Attribution 4.0 International license.

We transiently transfected HeLa cells displaying CD80-anchored GFP, leading to a heterogeneous population of GFP-positive and -negative cells. We then mixed in E. coli with a high surface density of VHH (E. coli VHH) with HeLa GFP whose respective adhesin and receptor were induced separately. After washing, we visualized the coculture by confocal microscopy. We observed that bacteria bound to GFP-positive HeLa but not to GFP-negative cells (Fig. 1B). This indicated that the synthetic system is specific, validating it as a model of bacterial adhesion. As a result, we generated a stable and clonal doxycycline-inducible HeLa GFP-display cell line (HeLa GFP) and grew cultures of this line in microchannels to investigate adhesion under flow conditions. We diluted bacteria in mammalian cell culture medium and loaded them on a syringe pump for flow control. We injected the bacterial suspension in the microchannel covered with HeLa GFP. After 1 h under moderate flow, we imaged cells in the channel by confocal microscopy and quantified the number of bacteria per mammalian cell. The bacterial counts per HeLa GFP cell were larger when both constructs were highly induced compared to uninduced or low-induction conditions (Fig. 1C). Preincubation of E. coli VHH with recombinant soluble GFP decreased the bacterial count per HeLa GFP cell back to the noninduced condition (Fig. 1C). Therefore, this system yields selective and dose-dependent bacterial adhesion of VHH-displaying bacteria to GFP-displaying HeLa cells under both static and flow conditions. Our initial characterization overall demonstrates that in tandem, E. coli VHH and HeLa GFP represent a realistic, tunable model for specific microbial adhesion to host mammalian cells.

### Bacteria attach to host cells in two successive steps.

Our initial results showed that the number of bacteria attached to host cells depends on the induction levels of both VHH adhesin and GFP receptor (Fig. 1B and C). We wondered whether this was due to changes in the number of bacteria attaching to or detaching from the host cell surface (Fig. 1D). This question motivated us to inspect the dynamics of attachment to HeLa cells at the single-bacterium level. We tracked attachment and detachment of single bacteria over the course of 1 h (see Movie S1 at https://doi.org/10.5281/zenodo.5079719). These visualizations helped us identify two classes of attachment behaviors. First, a large proportion of bacteria were visible only on single frames, indicating that they were in contact with the membrane for a few seconds. Another population of cells stayed attached for much longer times. We were intrigued by this dichotomy in adhesion behaviors and performed multiscale imaging to characterize each step.

To inspect short-timescale attachment events, we performed fast confocal imaging of attachment (1 frame/s). We found that a large proportion of bacteria stayed on the membrane for only about 2 s (one or two frames; see Movie S2 at https://doi.org/10.5281/zenodo.5079719). We then quantified the proportion of bacteria that attached to the host surface for more than 2 s relative to the total number of contacts, which we call contact efficiency (Fig. 1E). We found that the contact efficiency was on average only 7% when both VHH and GFP were induced. We then compared this contact efficiency between adhesin-receptor conditions. Surprisingly, we found that neither the presence of VHH adhesins nor that of GFP receptors influenced the contact efficiency (Fig. 1E). This suggests that this early stage is not specific.

We thus speculated that the adhesin-receptor interactions regulate bacterial attachment on a longer timescale. To test this hypothesis, we timed single bacteria residing the surface of host cells during a 1-h-long movie (see Movie S1 at https://doi.org/10.5281/zenodo.5079719). We thus built inverse cumulative residence time distributions (Fig. 1F). We found that these distributions had exponential-like decays, which we could fit to the sum of two exponential functions (Fig. 1F and Materials and Methods). This highlighted two characteristic timescales over which bacteria detached from the surface. The shortest timescale is on the order of 100 s and was nearly identical between conditions (Fig. 1G). The longest timescale τ_res_, associated with the second exponential, showed large variations between VHH and GFP configurations (Fig. 1H). We measured a 10-fold increase in τ_res_ when bacteria displayed a high VHH density compared to bacteria displaying an empty intimin scaffold (no VHH). In addition, we measured a 3.5-fold decrease when we did not induce GFP on HeLa cells. These results imply that adhesin-receptor interactions materialize only over minutes. As a comparison to typical association rates, we estimated the on- and off-rates of adhesin-ligand based on known kinetics constants of VHH-GFP (30). Interestingly, the off-rate of VHH reflects a characteristic time of 6,900 s, which is of the same order of magnitude as our τ_res_ measurements. For an arbitrary GFP concentration of 1 μM, the on-rate yields a reaction time on the order of 1 s, 2 orders of magnitude shorter than our measurements. This suggests that other factors mediate the first adhesion step, before adhesins engage with their ligand. In summary, we highlighted that bacteria specifically attach to host cells by going through an initial nonspecific attachment followed by adhesin-receptor docking, thereby promoting long-lasting physical contact.

We then tested the contributions of biochemical properties of the adhesin in regulating attachment. We swapped the adhesin to two other VHH sequences coding for anti-GFP nanobodies of different affinities (*K_D_* [equilibrium dissociation constant]) and kinetic rates (*k*_on_ and *k*_off_) (31). We checked that their expression levels were unaffected using anti-HA fluorescein isothiocyanate (FITC)-labeled antibodies (Fig. S2A to C, i). We first verified that the fusion to intimin did not affect *K_D_*. Titrating these alternate VHH forms on E. coli with GFP yielded *K_D_*s matching their *in vitro* measurements performed with soluble recombinant proteins (Fig. S2A to C, ii and iii) (30, 31). We thus performed adhesion experiments on HeLa GFP under flow with E. coli expressing the alternate VHH forms. We observed a slight positive correlation between bacterial load per HeLa cell and VHH affinity across 3 orders of magnitude of *K_D_* and 2 orders of magnitude of *k*_off_ (Fig. S2A to D). Consistent with its nonspecific nature, the contact efficiency was independent of the affinity of the nanobody to GFP (Fig. S2E). On the longer timescale, we measured higher τ_res_ and a statistically significant increase in the preexponential factor *C*_res_ at higher affinities (Fig. S2F and G), explaining the differences in the bacterial load. Altogether, the dependence of the specific adhesion step on adhesin biochemistry was surprisingly weak compared to the changes induced by adhesin expression levels (Fig. 1H and Fig. S2F).

10.1128/mBio.01392-21.3 FIG S2 Functional validation and bacterial attachment as a function of VHH affinity to GFP. (A to C) We displayed and compared VHH with two different anti-GFP nanobodies (LaG02 and LaG94-10) at the surface of E. coli. These have distinct biochemical properties including affinity (*K_D_*) and on- and off-rates. (i) Their level of expression was measured by HA staining. Bar, 1 μm. (ii) Their functionality was then assessed by staining with an excess amount of recombinant GFP. (iii) By titrating with recombinant GFP, we could determine the *K_D_* of displayed nanobodies. These remain in the range of the published values obtained with soluble recombinant nanobodies. Bacteria were induced with 250 ng/ml tetracycline overnight prior to staining. Error bars represent standard deviation from triplicate fields. The solid line represents a function fit as described in Text S1. Image intensity scale is identical between samples. (D) Final E. coli-nanobody count per HeLa cell positively correlates with nanobody affinity in flow. Nanobody display induction level is high. (E) The contact efficiency does not depend on the nanobody affinity to GFP. (F) Comparison of the characteristic residence time τ_res_ as a function of nanobody affinity. An outlier of value 775,000 s was excluded from further analysis. (G) Higher affinity increases the proportion of bacteria irreversibly binding to cells. Statistical tests: one-way ANOVA followed by Tukey *post hoc* test (**, *P* < 0.01; *, *P* < 0.05). Download FIG S2, TIF file, 0.7 MB. Copyright © 2021 Pierrat et al. 2021 Pierrat et al. https://creativecommons.org/licenses/by/4.0/ This content is distributed under the terms of the Creative Commons Attribution 4.0 International license.

### Bacteria attach to abiotic surfaces in a single specific step.

**FIG 2:**
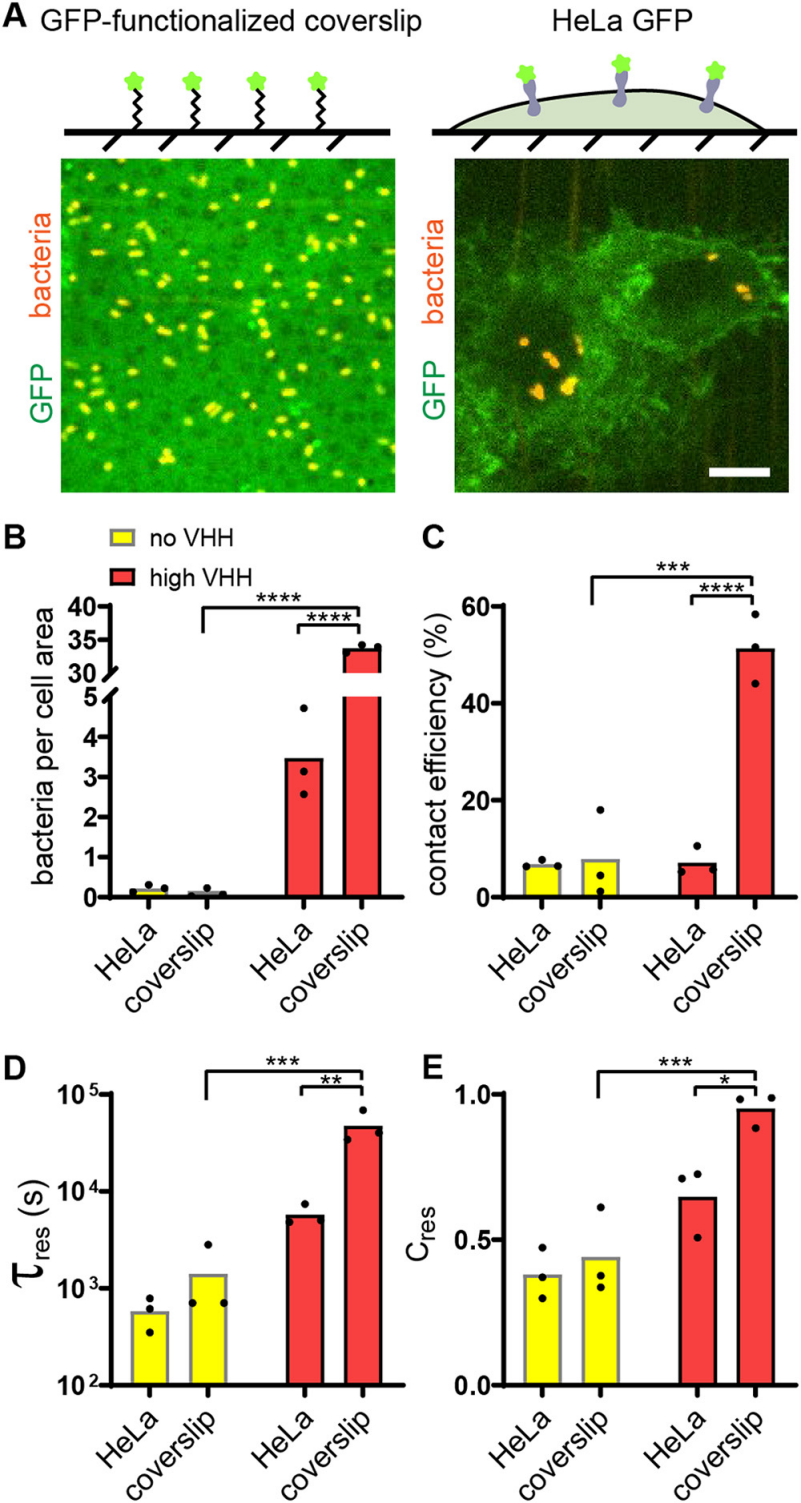
Attachment of bacteria to abiotic surface is a single-step process. (A) (Top) Controlled GFP-functionalized coverslips permit visualization of specific adhesion to hard, abiotic surface and quantitative comparison with adhesion to mammalian cells. (Bottom) Representative confocal microscopy images of bacterial binding to GFP-coated coverslips (left) and HeLa-GFP (right). Bar, 10 μm. (B) Final bacterial count per cell area is about 10-fold larger on GFP-coated coverslips than on HeLa cells in the presence of VHH. (C) Bacterial contact efficiency is higher on GFP-coated coverslips than on HeLa cells in the presence of VHH. (D) The characteristic residence time τ_res_ shows the VHH-dependent binding to coverslips is stronger than that to HeLa cells. (E) Relative contribution of short- and long-timescale exponential fits shows that 95% of E. coli VHH bacteria strongly bind to GFP-coated coverslips. Statistical tests: two-way ANOVA and Sidak *post hoc* test (****, *P* < 10^−4^; ***, *P* < 0.001; **, *P* < 0.01; *, *P* < 0.05).

We suspected that the complex of physical microenvironments of the host cell membrane plays a role in either of the two successive steps of attachment. To provide additional insights on these factors, we compared the specific adhesion of E. coli to the surface of an abiotic material with the one on mammalian cells (Fig. 2A). We engineered specific adhesion to glass by conjugating receptors to a coverslip substrate. We conjugated N-terminally His-tagged recombinant GFP to nitrilotriacetic acid (Ni-NTA) functionalized glass, on which we bonded elastomeric microfluidic channels (see Materials and Methods). We monitored the dynamics of specific adhesion to abiotic surface by flowing a bacterial suspension in the GFP-coated microchannel. We observed bacteria almost exclusively attaching to the GFP-coated areas, thereby validating adhesion specificity (Fig. S3A to C and also Movie S3 at https://doi.org/10.5281/zenodo.5079719). These experiments highlighted a blatant difference from mammalian cells: there were 10 times more bacteria attached to the GFP-coated glass surface than on HeLa cells (Fig. 2B). This difference was strictly dependent on VHH-GFP interactions as bacteria only sparsely attached to untreated glass or to glass coated with mKate2, a red fluorescent protein that does not bind VHH (Fig. S3D).

10.1128/mBio.01392-21.4 FIG S3 Specificity of adhesion of E. coli-VHH to GFP-coated glass coverslips. (A) Microscopy image of the edge of a GFP-coated region on the coverslip. (B) Maximum-intensity projection of the corresponding field showing bacterial attachment (red) after 10 min in flow. (C) Overlay of panels A and B. (D) Maximum-intensity projection of E. coli VHH (red) after 60 min in flow on mKate2-coated coverslips. Bars, 10 μm. Download FIG S3, TIF file, 1.8 MB. Copyright © 2021 Pierrat et al. 2021 Pierrat et al. https://creativecommons.org/licenses/by/4.0/ This content is distributed under the terms of the Creative Commons Attribution 4.0 International license.

To further characterize the pronounced difference in adhesion between abiotic and biotic surfaces, we focused on attachment/detachment dynamics. We compared the early contact efficiencies and residence times of bacteria on glass with the ones on HeLa cells. First, we found that about 50% of E. coli VHH stayed attached to the GFP-coated glass surface upon initial contact, in contrast with the 7% of bacteria remaining on HeLa GFP cells (Fig. 2C). This largely contributed to the differences in bacterial accumulation at the end of the experiment. In addition, the characteristic residence time of E. coli VHH on glass was more than eight times longer than that on HeLa cells (Fig. 2D). This characteristic time was also much longer than the duration of our visualizations so that most bacteria can be considered irreversibly attached to glass. Finally, on the longer timescale, very few bacteria transiently bound to coverslips, as highlighted by the relative contribution of τ_transient_ (Fig. 2E). This further supports a scenario where adhesin and receptor engage rapidly and efficiently when an abiotic surface supports the receptors.

In summary, specific adhesion to an abiotic surface is controlled by early attachment events within the first few seconds of surface encounter, consistent with *in vitro* reaction rates. Successful attachment beyond this step leads to nearly irreversible surface association. Thus, a single specific step mediates attachment on abiotic surfaces, while phenomena at both short and long timescales regulate specific attachment to host cells.

### Host cell membrane mechanics regulate bacterial adhesion.

Given the differences in material properties between inert and living substrates, we hypothesized that the mechanical microenvironment of host cells may play a key role in regulating attachment. Following this intuition, we investigated the role of cell mechanics in the process of adhesion to host cells. Host cell mechanics depend on the intrinsic membrane bilayer properties but also on emergent properties provided by the actin cytoskeleton.

**FIG 3:**
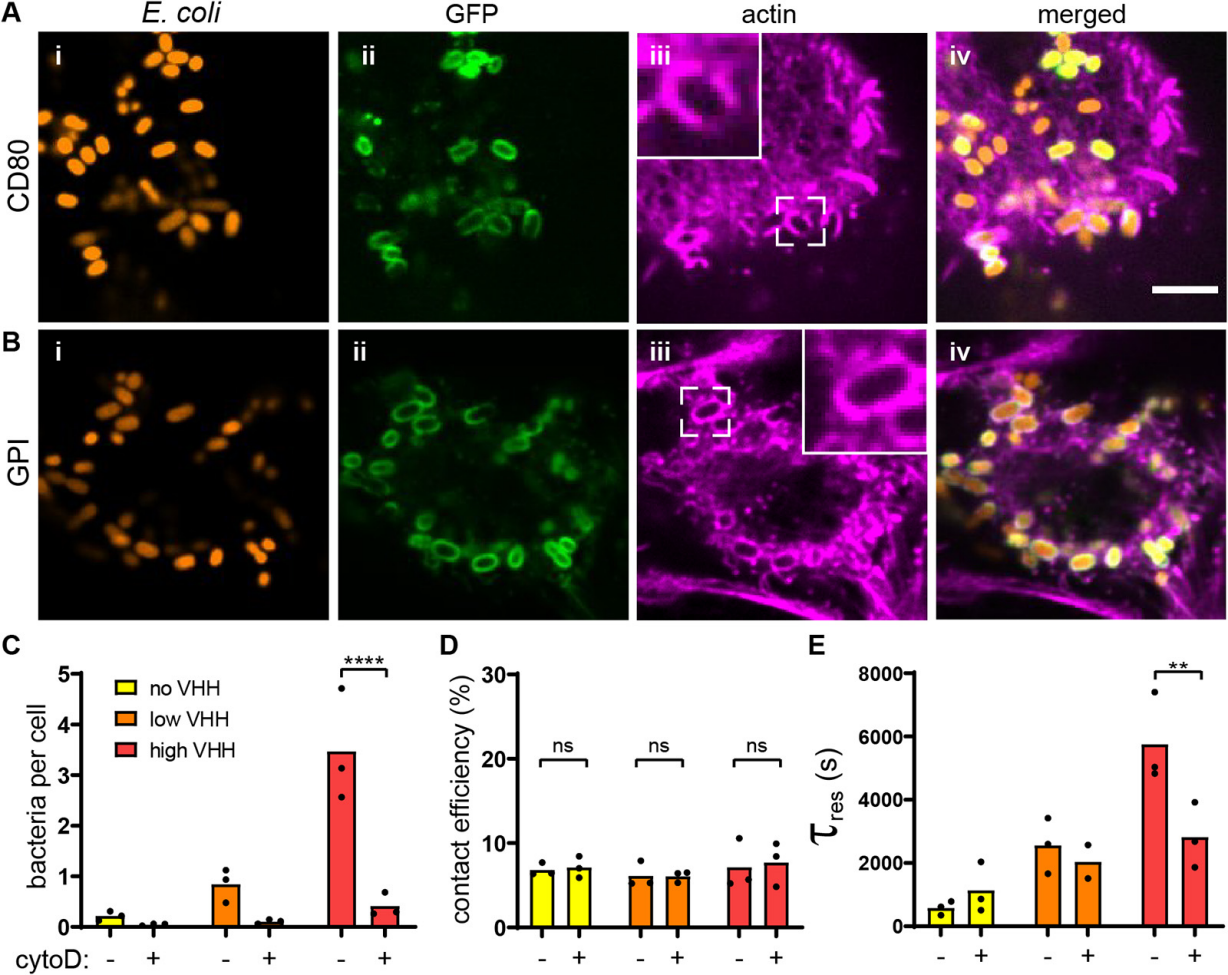
Regulation of bacterial adhesion by host cytoskeleton. (A) Actin rearranges around attached bacteria. After static incubation with E. coli VHH (orange), HeLa cells displaying GFP with a CD80 anchor (green) were stained for actin (purple). Bar, 5 μm. (B) Bacteria promote actin embeddings in the absence of any cytosolic component in the mammalian cell. After static coculture with E. coli VHH (red), HeLa cells displaying GFP with a glycosylphosphatidylinositol (GPI), which does not harbor any cytosolic signaling domain, also show strong actin remodeling around attached bacteria. (C) HeLa cell treatment with the actin polymerization inhibitor cytochalasin D (cytoD) reduces the bacterial count per HeLa cell. (D) Bacterial contact efficiency is independent of actin polymerization. (E) The characteristic residence time τ_res_ decreases in the presence of cytochalasin D at high VHH density. Statistical tests: two-way ANOVA and Sidak *post hoc* test (****, *P* < 10^−4^; **, *P* < 0.01; ns, not significant).

We observed that bacteria attached to HeLa cells accumulate GFP at their surface, as if they were embedded into membrane invaginations (Fig. 3A, i and ii). Given the role of the cytoskeleton in the shape and mechanics of eukaryotic cell membranes, we hypothesized that actin could play a role in bacterial attachment. To first explore this possibility, we visualized the actin cytoskeleton of bacterium-bound cells using fluorescent phalloidin staining. The actin density increased around individual attached bacteria, indicating a potential morphological remodeling of the membrane upon attachment (Fig. 3A, iii and iv). Our GFP display construct is based on a truncation of the CD80 receptor that is overexpressed in macrophages with notoriously increased actin remodeling. To exclude the possibility that remodeling is an artifact of the C-terminal CD80 anchor, we fused GFP to a glycosylphosphatidylinositol (GPI) membrane anchor devoid of cytosolic signaling components (Fig. S1C) (32). There, we could also observe a similar actin remodeling and membrane surrounding bacteria (Fig. 3B and Fig. S4A and B). The membrane remodeling occurred within minutes, on a similar timescale as the GFP uptake (see Movies S4 and S5 at https://doi.org/10.5281/zenodo.5079719 and Fig. S4C and D). Actin-dependent membrane remodeling could thus increase the contact area between bacteria and host cell, stimulating adhesin-receptor interactions and consequently increasing adhesion strength.

10.1128/mBio.01392-21.5 FIG S4 HeLa cells actively remodel plasma membrane around bacteria. (A and B) Merged three-dimensional (3D) visualization of a confocal z-stack after 1 h of E. coli VHH (red) or HeLa cells displaying GFP with a GPI anchor (green) in static coculture. Actin was stained with phalloidin (pink), and nuclei with DAPI (blue). Events of actin remodeling are indicated by white arrows in panel B. Bar, 10 μm. (C) HeLa GFP (CD80) cells actively pull bacteria toward their cell body. Maximum-intensity projection of a confocal time-lapse experiment of E. coli VHH (red), HeLa GFP (green), and flow, corresponding to Movie S4 at https://doi.org/10.5281/zenodo.5079719. Bar, 5 μm. (D) Bacteria sequester GFP within minutes under static conditions. Quantification of the mean fluorescence of *n* = 11 bacteria in Movie S5 at https://doi.org/10.5281/zenodo.5079719. Error bars represent the standard error. Download FIG S4, TIF file, 1.9 MB. Copyright © 2021 Pierrat et al. 2021 Pierrat et al. https://creativecommons.org/licenses/by/4.0/ This content is distributed under the terms of the Creative Commons Attribution 4.0 International license.

We further tested the role of membrane remodeling in bacterial attachment by employing cytochalasin D (cytoD), a drug inhibiting actin polymerization (33). We measured an 8-fold reduction in E. coli VHH attachment on treated cells compared to the untreated control (Fig. 3C). Inhibiting actin polymerization did not decrease the contact efficiency of bacteria at early timescales (Fig. 3D). However, bacterial residence time was decreased in the presence of the drug (Fig. 3E). This difference was most dramatic for higher VHH densities. This suggests that membrane remodeling upon attachment takes place on the minute timescale, thereby stabilizing adhesin-receptor interactions.

### The glycocalyx shields the host from receptor-specific bacterial adhesion.

Membrane mechanics regulate how bacteria engage in specific adhesion to host cells on timescales of minutes. Still, membrane mechanical properties had little effect on the nonspecific adhesion step, which differed so much between glass and cells, as the contact efficiencies upon membrane and cytoskeletal perturbations remained below 10% (Fig. 3D). We thus still wondered why such a small proportion of bacteria could commit to specific adhesion upon encountering the host cell surface.

**FIG 4:**
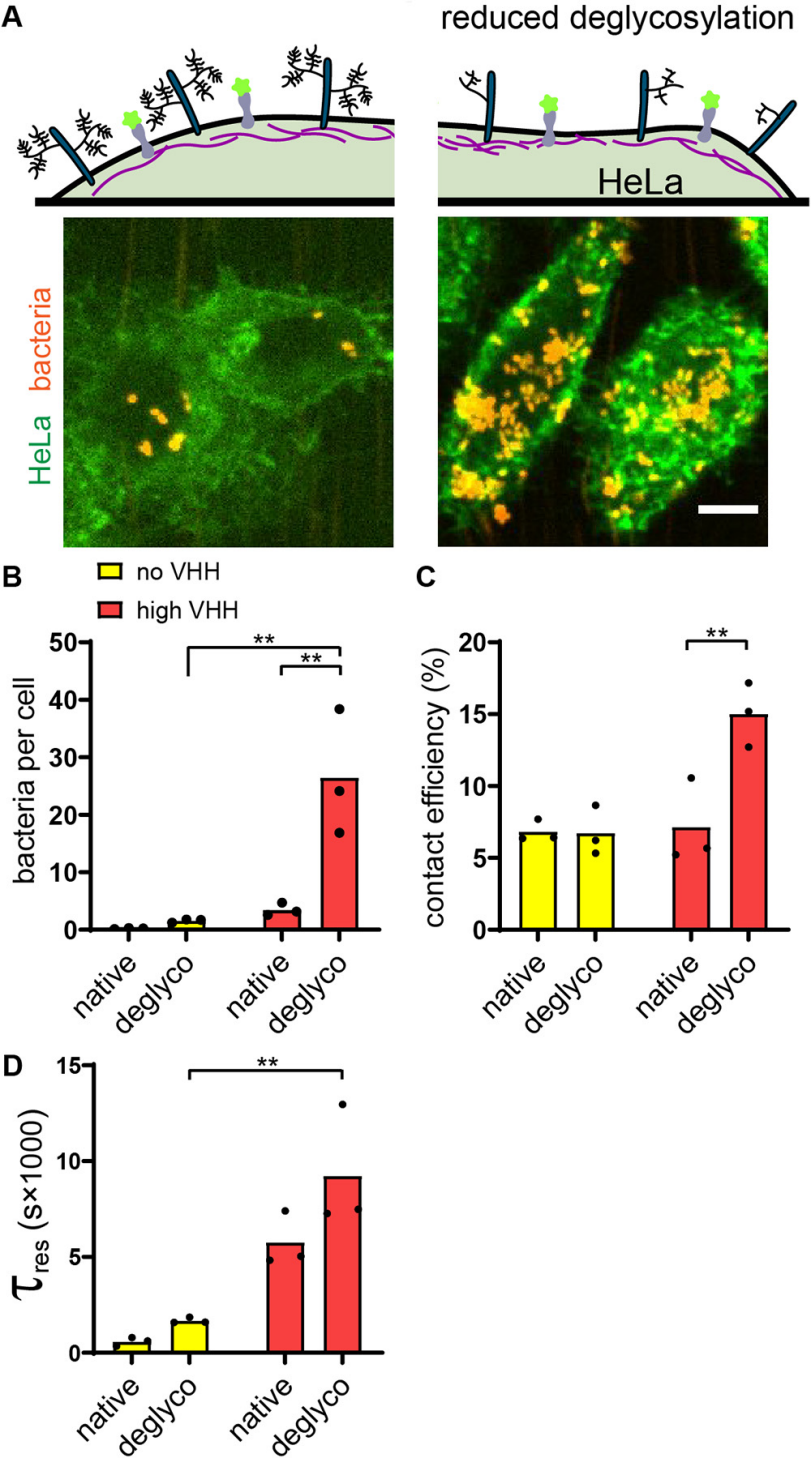
The membrane glycocalyx inhibits bacterial attachment. (A) Enzymatic deglycosylation of HeLa cell surface proteins increases bacterial binding. The right image shows two deglycosylated HeLa cells covered by E. coli VHH while the negative control under otherwise identical conditions has a low bacterial count. Bar, 10 μm. (B to D) Comparison of bacterial adhesion dynamics between untreated cells (native) and deglycosylated cells (deglyco). (B) Final E. coli VHH count per HeLa cell is higher in deglycosylated cells. (C) Glycocalyx removal increases the contact efficiency of E. coli VHH. (D) Comparison of the characteristic residence time τ_res_ with or without deglycosylation mix. Statistical tests: two-way ANOVA and Sidak *post hoc* test (**, *P* < 0.01).

We reasoned that other mechanical components of the host cell surface could play a role in limiting bacterial adhesion. We thus hypothesized that the glycocalyx, a dense layer of glycoproteins and glycolipids that decorates the surface of most mammalian cells, could limit attachment. To test this, we investigated the role of the host glycocalyx in the dynamics of bacterial adhesion. We cultured HeLa GFP cells with a deglycosylating mix of enzymes, thereby promoting its degradation (Fig. 4A) (34, 35). We confirmed specific enzymatic activity in mammalian medium by digesting fetuin, an N- and O-glycosylated control protein (Fig. S5A). We also stained HeLa cells with rhodamine-labeled wheat germ agglutinin and observed a decrease in fluorescence in cells treated with the deglycosylating mix of enzymes (Fig. S5B). We then tracked bacterial adhesion dynamics at the surface of deglycosylated HeLa cells, which showed a dramatic effect. First, there were six times more bacteria attached to deglycosylated cells compared to their native, untreated state (Fig. 4B). The bacterial density on deglycosylated cells reached values close to the ones measured on glass (Fig. 2B). We further examined the specific contributions of the glycocalyx in attachment dynamics by comparing contact efficiency and residence time distributions to the native state. Consistent with our hypothesis, we found that bacteria remained attached twice as efficiently to deglycosylated cells as to untreated cells in a VHH-dependent manner (Fig. 4C). Deglycosylation only slightly increased the characteristic residence time, in both the presence and absence of VHH (Fig. 4D). Altogether, our data indicate that the mammalian glycocalyx shields the host cell membrane from direct engagement of bacterial adhesins to target receptors, thereby nonspecifically limiting bacterial attachment.

10.1128/mBio.01392-21.6 FIG S5 Deglycosylation activity in mammalian cell culture medium. (A) SDS-PAGE gel showing 10 μg of fetuin incubated overnight at 37°C in 20 μl with either only 1 μl peptide-*N*-glycosidase F (PNGase) or 1 μl of the deglycosylation mix of enzymes targeting N- and O-glycosylated proteins in either the nondenaturing buffer provided by the supplier (Buf) or FluoroBrite medium (Med) supplemented with GlutaMAX. The gel indicates partial deglycosylation of fetuin under mammalian cell culture conditions compared to untreated fetuin (ctrl) and compared to ideal buffer conditions. (B) Live HeLa cells treated with the deglycosylation mix of enzymes show a reduction in *N*-acetylglucosamine staining with wheat germ agglutinin (WGA). For WGA, we display inverted pictures of maximum-intensity projection from confocal stacks. Bar, 40 μm. Download FIG S5, TIF file, 1.8 MB. Copyright © 2021 Pierrat et al. 2021 Pierrat et al. https://creativecommons.org/licenses/by/4.0/ This content is distributed under the terms of the Creative Commons Attribution 4.0 International license.

Since our system uses a truncated intimin, we generated a full-length fusion to test whether an extended scaffold could help overcome the glycocalyx. Bacterial load per cell decreased for the extended linker, as a result of a decrease in the characteristic residence time (Fig. S6A and B) but had surprisingly no effect on contact efficiency (Fig. S6C). One explanation for this would be that the longer linker would directly be responsible for the decrease in the characteristic residence. Alternatively, the fusion could be expressed to different levels, causing a change in the second step (Fig. 1H). Staining the extended intimin-VHH fusion with recombinant enhanced GFP (eGFP) showed a 2.5-fold reduction in GFP signal compared to the truncated form, indicating that reduced expression participates in shortening residence time and weakening adhesion (Fig. S6D and E). In summary, linkers longer by a few nanometers do not help overcome the glycocalyx barrier.

10.1128/mBio.01392-21.7 FIG S6 Longer VHH linker does not improve binding in flow. (A) Final E. coli VHH count per HeLa cell is reduced with full-length intimin compared to truncated intimin at high and low levels of induction. (B) The characteristic residence time is reduced with full-length intimin at a high level of induction. (C) Linker size does not affect the contact efficiency. (D and E) Staining of “high-VHH” bacteria using recombinant eGFP highlights a 2.5-fold decrease in the surface display efficiency. Statistical tests: one-way ANOVA and Tukey *post hoc* test (*, *P* < 0.05; ****, *P* < 10^−4^). Download FIG S6, TIF file, 1.8 MB. Copyright © 2021 Pierrat et al. 2021 Pierrat et al. https://creativecommons.org/licenses/by/4.0/ This content is distributed under the terms of the Creative Commons Attribution 4.0 International license.

### Flagella and flow counteract the glycocalyx shield.

**FIG 5:**
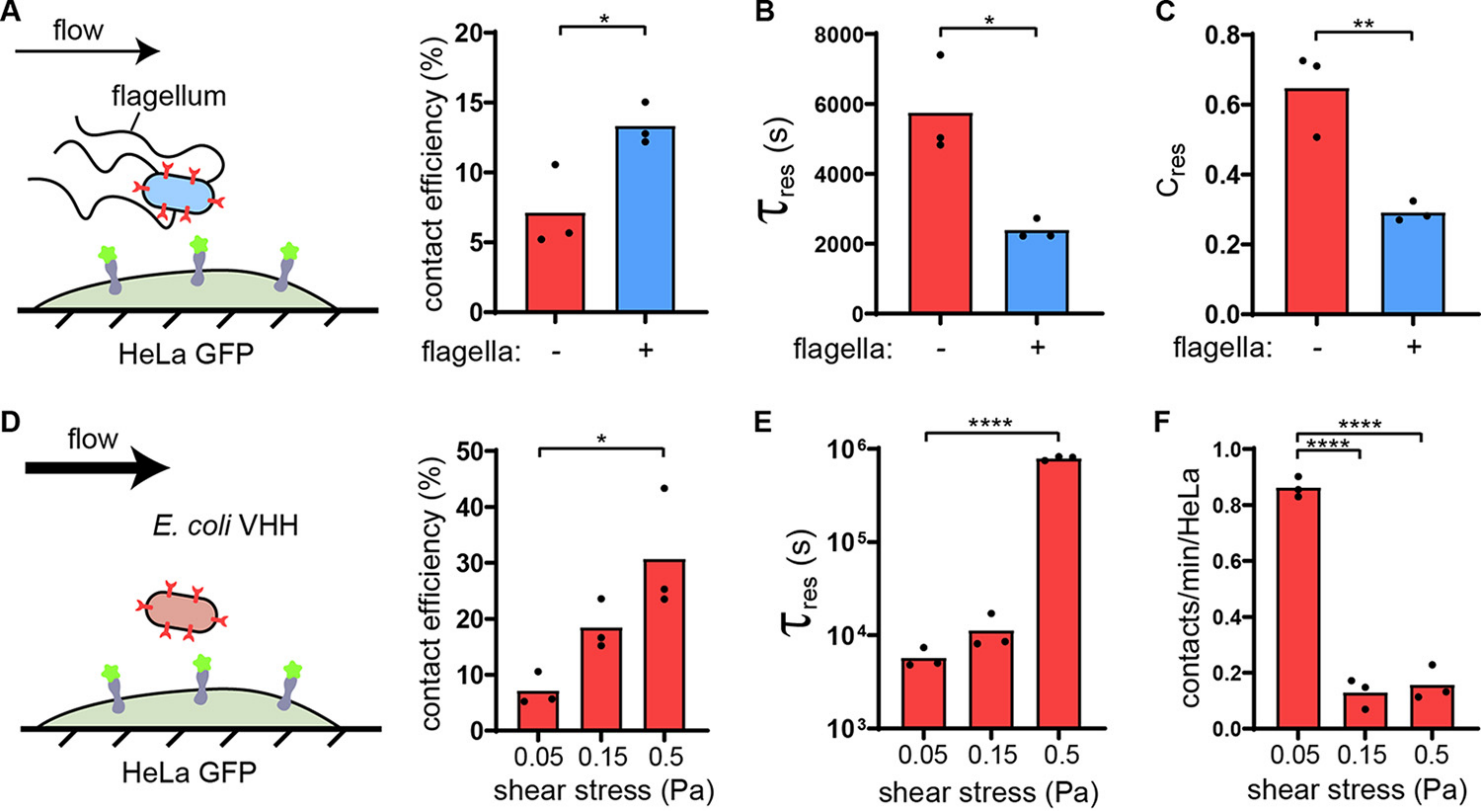
Flagella and flow attenuate the glycocalyx shield. (A) Schematic of the experimental setup. Flagellated E. coli VHH (blue) was compared to nonflagellated E. coli VHH (“+” and “−,” respectively). E. coli VHH contact efficiency is increased in the presence of flagellum in flow. (B) The presence of flagella decreases the characteristic residence time in flow. (C) Comparison of the preexponential factor of the characteristic transient binding time τ_transient_ in the presence or absence of flagella shows that the proportion of bacteria strongly binding to HeLa GFP is lower with flagella. (D) Schematic of the experimental setup. We measured the attachment dynamics of E. coli VHH in increasing shear stresses. Bacterial contact efficiency increases with flow intensity. (E) The characteristic residence time τ_res_ increases with flow intensity. (F) Strong flows decrease the contact frequency despite a higher number of bacteria crossing the channel. Statistical tests for panels A to C: two-tailed unpaired *t* test (**, *P* < 0.01; *, *P* < 0.05). Statistical tests for panels D to F: one-way ANOVA and Tukey *post hoc* test (*, *P* < 0.05; ****, *P* < 10^−4^).

Beyond simple short-range adhesins such as the ones belonging to the class of autotransporters, bacteria often display surface extensions such as flagella and fimbriae, sometimes capped with adhesins. These extended structures could help overcome the physical glycocalyx barrier by reaching through, thereby promoting the first step of adhesion. We thus explored how surface filaments could play a role in the early adhesion step. We first compared the binding of flagellated and nonflagellated bacteria to HeLa GFP. We could not distinguish the bacterial numbers between flagellated and nonflagellated strains at the end of the experiments (Table S2). However, the details of attachment dynamics revealed that the flagellum mediates a trade-off between nonspecific and specific adhesion. On the one hand, we observed that flagellated E. coli has higher contact efficiency (Fig. 5A). This shows that flagella promote short-timescale nonspecific attachment. On the other hand, the characteristic residence time of flagellated E. coli was more than twice as short as its nonflagellated counterpart (Fig. 5B). Consistent with this, the transient characteristic residence time was similar between conditions but the preexponent factor *C*_res_ had significantly decreased weight in our exponential fits, reflecting a high number of bacteria transiently binding and fewer bacteria strongly binding (Fig. 5C and see Movie S6 at https://doi.org/10.5281/zenodo.5079719). Altogether, flagella mediate a trade-off in adhesion, increasing early commitment while decreasing subsequent specific attachment.

10.1128/mBio.01392-21.9 TABLE S2 Raw data of all parameters of the study. Download Table S2, XLSX file, 0.02 MB. Copyright © 2021 Pierrat et al. 2021 Pierrat et al. https://creativecommons.org/licenses/by/4.0/ This content is distributed under the terms of the Creative Commons Attribution 4.0 International license.

Finally, we wondered whether fluid flow could balance the effect of the glycocalyx. Typically, hydrodynamic forces positively select for single bacteria whose adhesion force exceeds shear force. In the context of adhesion to host cells and based on molecular dynamic simulations, we suspected that flow could generate a shear force that deforms the ∼100-nm-thick glycoprotein layer, thereby reducing shielding (36). Given that these two flow-induced effects are antagonistic, we wondered how their combined contributions would ultimately affect bacterial attachment. We thus performed experiments with adhesion of E. coli VHH to HeLa GFP under three different flow regimes. We applied flow rates that generated shear stress of 0.05, 0.15, and 0.5 Pa at the channel centerline. These stresses generate 0.1, 0.3, and 1 pN hydrodynamic forces on single bacteria, respectively (assuming a bacterium is 2 μm long and 1 μm wide) (37). We measured contact efficiency and residence times, which are normalized metrics, i.e., they do not depend on the influx of bacteria in the channel.

The contact efficiencies increased with shear stress, from 7% at low shear up to 31% at high shear (Fig. 5D). This indicates that flow promotes the nonspecific adhesion within the few seconds after contact. On the timescale of minutes where adhesins engage their GFP receptors, the characteristic residence times of bacteria increased strongly with shear stress, up to 2 orders of magnitude (Fig. 5E). Despite longer residence time and higher contact efficiency in strong flow, we could not measure clear changes in absolute bacterial load per HeLa cell compared to weaker flows (Table S2). We could attribute this to an unexpected decrease in the absolute number of bacterial contacts per mammalian cell with increasing flows, indicating that bacteria are less likely to encounter the host cell membrane under strong shear (Fig. 5F). Altogether, our results suggest that higher flows improve bacterial attachment in two ways. First, stronger flow promotes early attachment by counteracting the glycocalyx. Second, increased flow further engages adhesins with their receptors.

## DISCUSSION

To infect or stably colonize their hosts, bacterial pathogens and commensals attach to the surface of biological tissues (38). Adhesins are the major ingredient of bacterial adhesion *in vivo*. By binding to target receptor moieties at the surface of host cells, they confer strong attachment and specificity. We investigated how bacteria adhere to host cells by leveraging a tunable synthetic system comprising an adhesin (VHH) and a receptor (GFP). This system had been engineered for therapeutic VHH library screening and has been applied to the study of multicellular self-organization of bacterial populations (26, 27). We here repurposed it to investigate bacterial attachment to host cells while controlling adhesin expression and binding strength without affecting host viability.

**FIG 6:**
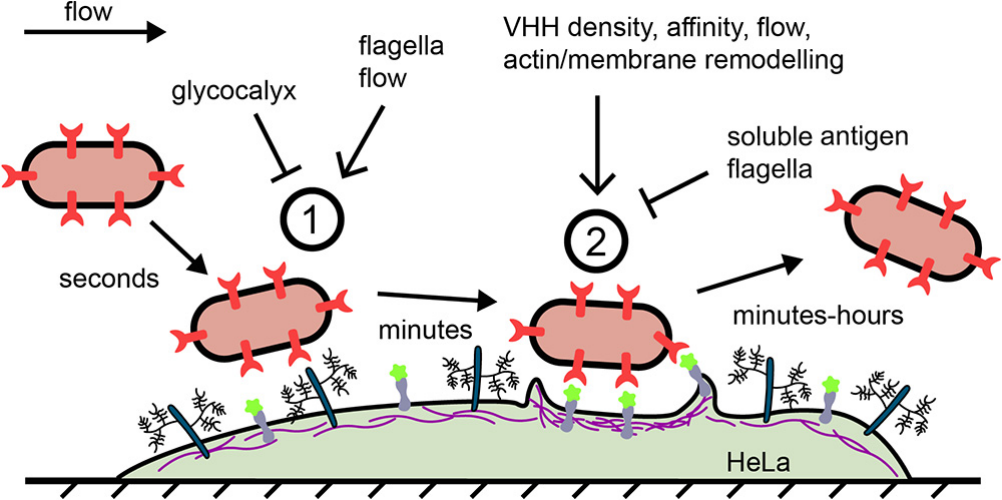
A model for mechanically-regulated, two-step bacterial attachment to host cells. Upon contact of a bacterium with a host cell (1), the glycocalyx blocks attachment by sterically shielding the membrane. This short-timescale interaction does not involve short-range adhesins or mammalian membrane receptors. Strong shear forces and bacterial flagellum can increase the transient binding efficiency, in part by attenuating the glycocalyx shield. The bacterium subsequently binds adhesins onto host receptors to promote specific adhesion (2). This increased adhesin density, affinity to the receptor ligand, flow, and actin polymerization promote the specific adhesion step, while the flagella and soluble antigen repress it, promoting bacterial detachment.

We leveraged the versatility of the VHH-GFP system to perform a careful investigation of the dynamics adhesion. We first identified a temporal aspect of bacterial attachment to host cells, where a two-step sequence leads to specific attachment (Fig. 6). After contact, bacteria attach nonspecifically to host cells for not more than a minute. Bacteria subsequently engage adhesins with their receptors on a timescale consistent with adhesin-ligand rupture kinetics, in our case for minutes to hours. Sequential adhesion to host cells contrasts with the single specific step governing adhesion to abiotic surfaces (Fig. 2). Bacterial adhesion has previously been characterized as a multistep process, be it on abiotic surfaces (reversible followed by irreversible during biofilm formation) or on host cells (sequential deployment of adhesins). Our results distinguish themselves from these other multistep processes as they involve a single adhesin and as host factors regulate each of these steps.

The VHH display system allowed us to test the contributions of adhesin density and binding kinetics in attachment. While specific attachment increased with VHH density, the adhesin affinity and reaction rates ended up being a surprisingly weak regulator of attachment and detachment. This could be explained by the fact that after engaging several adhesins of relatively high affinity, bacterial overall avidity rapidly predominates over the affinity of individual adhesins (39). In contrast, we found that mechanical factors of the host environment strongly regulate each of the stages of adhesion. The host glycocalyx, a layer of glycans bound to glycolipids and surface glycoproteins, inhibits the first adhesion step by physically shielding the host membrane surface; in the case, it is not the target itself (38, 40). Then, we found that the host cell actin cytoskeleton shapes the membrane around attached bacteria, thereby improving specific adhesion. Membrane-embedded bacteria could thus engage VHH with additional GFP receptors, increasing overall adhesion strength. We propose that a passive ratchet mechanism triggers the actin-dependent membrane encapsulation of bacteria (41, 42).

Surprisingly, we found that fluid flow improved attachment of E. coli VHH to HeLa GFP, during both nonspecific and specific stages of adhesion. This was unexpected because fluid flow, by virtue of the shear force it generates, tends to remove bacteria from their attachment surface (37). By shearing the glycocalyx, flow could improve the access of the bacterium to the cell membrane, thereby increasing nonspecific contact efficiency (36). Concerning the subsequent specific step, our observations are reminiscent of flow-enhanced adhesion as a result of the formation of catch bonds, as in streptococci and uropathogenic E. coli (43, 44). However, VHH-GFP do not form catch bonds at the molecular level (45). We hypothesize that flow improves specific adhesion via an indirect mechanism. For example, shearing of a bound bacterium generates tension onto the membrane, thereby stimulating actin recruitment (46). This in turn engages more receptors, ultimately strengthening attachment. We finally note that as shear stress increases, more stringent selection for strongly attached cells could lead to the observed enhanced attachment. As a result, we cannot rule out that shear removes loosely attached bacteria at a rate that is higher than the temporal resolution of our imaging. All things considered, we demonstrated that the dependence of bacterial attachment on hydrodynamic forces cannot be simply extrapolated from a physically simplified behavior of a bacterium attached to a hard, inert surface.

By engineering autotransporter-based adhesins, we could model a single type of adhesins. However, our results bring a new perspective on other adhesin types in the context of infection. We specifically highlighted the regulatory role of the glycocalyx in early attachment. Pathogens may overcome this first barrier using different strategies. For example, Salmonella uses reversible and irreversible sets of adhesin and actively degrades the glycocalyx during infection, strengthening attachment (25, 47). Another strategy consists of adhering to the glycocalyx directly rather than to membrane proteins or to overcome the glycocalyx with adhesins that cap long pili or fimbriae. This in principle improves the efficiency of the first step of attachment, but it usually needs to be combined with subsequent adhesive processes for tighter contact with the cell membrane (5, 25, 40). Thus, adhesins targeting glycans and pilus-associated adhesin could bind more efficiently in the first step, but cytoskeleton-dependent adhesion reinforcement would be limited in the second (44, 48, 49).

During the process of infection, bacteria use an arsenal of virulence factors. These are deployed in a timely fashion in response to relevant signals. Synchronizing expression of virulence factors with host cell contact could promote timely deployment (37). For example, enteropathogenic E. coli transfers the intimin adhesin receptor Tir to gut epithelial cells upon contact (2). The nonspecific first step of adhesion thus offers a window of opportunity to deploy these systems within minutes.

Altogether, we have demonstrated that bacterial attachment to host cells differs from the expected behavior of simple adhesin-receptor interactions. Adhesin biochemistry and the physics of adhesion to inert materials only poorly predict adhesion to mammalian cells. This has therefore important implications in our view of infection. In the current context of the rise of multidrug-resistant pathogens, our work provides new insights that could inform the development of antiadhesive therapeutics (3, 40, 50).

## MATERIALS AND METHODS

### Cloning.

Plasmid cloning strategy and primer sequences are described in Table S1 in the supplemental material. Cloning was performed by restriction enzymes (NEB) and ligation with T4 ligase (Bioconcept) or by Hi-Fi Gibson assembly (NEB). PCRs were performed using Phusion polymerase (Life Technologies) and DNA purification with commercially available kits. Chemically competent XL10Gold (Agilent) was used for transformation.

10.1128/mBio.01392-21.8 TABLE S1 Plasmid cloning strategy and primers. Download Table S1, DOCX file, 0.02 MB. Copyright © 2021 Pierrat et al. 2021 Pierrat et al. https://creativecommons.org/licenses/by/4.0/ This content is distributed under the terms of the Creative Commons Attribution 4.0 International license.

### Cell culture, engineering, and induction.

HeLa cells were cultured in Dulbecco modified Eagle medium (DMEM) (ThermoFisher) supplemented with 10% fetal bovine serum (FBS) (Life Technologies) at 37°C and 5% CO_2_. Prior to experiments, cells were trypsinized and resuspended in FluoroBrite (Life Technologies) supplemented with 10% FBS and 1% GlutaMAX (Life Technologies). Cells were seeded at 100,000 cells/ml in 96-well plates or 400,000 cells/ml in microchannels (Ibidi μ-Slide VI 0.4) 1 day prior to experiments. In microchannels, first 30 μl of cell suspension was added. Cells were left to adhere for 5 to 6 h, and then reservoirs were filled with an additional 120 μl of medium.

Unless stated otherwise, we used HeLa cells displaying a doxycycline-inducible truncated CD80-anchored GFP. To generate a stable cell line, we produced lentiviruses in HEK293T cells. Cells at 50% confluence were cotransfected with pMD2G (Addgene 12259), pCMVR8.74 (Addgene 22036), and a lentivector encoding the doxycycline-inducible CD80-GFP display (pXP340, Table S1) using Lipofectamine 3000 (Life Technologies). Medium was changed at day 1, and lentiviruses were collected at days 2 and 3, separated from cell debris by centrifugation, sterile filtered, and added to HeLa cells. Cells were selected with G418 (Chemie Brunschwig) at 300 μg/ml, and resistant clones were obtained by limiting dilution in 96-well plates. The resulting monoclonal cell line (HeLa GFP) was induced overnight with doxycycline (HiMedia) at 300 ng/ml.

HeLa cells transiently expressing GPI-anchored GFP were obtained by lipofection of the plasmid PeGFP_GPI.

### Bacterial culture, engineering, and induction.

E. coli K-12 (BW25113) was cultured in LB at 37°C. Bacteria were stably engineered to express cytoplasmic mScarlet using pZA002 for Tn*7* insertion (51). pZA002 consists in a synthetic constitutive promoter upstream of mScarlet ligated into pGRG36 for chromosomal integration. Deletion of the flagellum was performed using the lambda red system and the PCR product using oXP851 oXP852 on E. coli genomic DNA to delete the *FliCDST* operon (Table S1) (52). Flagellated and nonflagellated fluorescent E. coli bacteria were then electroporated with tetracycline-inducible intimin-based display constructs. pXP383 coding for the display of VHH of medium affinity was used in this study in nonflagellated E. coli unless stated otherwise. pXP384 and pXP388 display the VHH of lower and higher affinities, and pDSG323 displays the empty scaffold and was selected with kanamycin (Sigma) at 50 μg/ml (27). To prepare adhesion experiments, early stationary precultures were diluted 1:3,000 and induced with sublethal doses of tetracycline (Sigma, 50 ng/ml for low VHH induction and 250 ng/ml for high VHH or for the empty intimin scaffold) overnight under shaking conditions.

### Cytoskeletal and glycocalyx perturbation.

Cytochalasin D (Sigma) at 1 μM was added 5 min prior to and during the experiment. One microliter of protein deglycosylation mix II (NEB) was added per channel for overnight treatment (150 μl total).

### Attachment with soluble GFP.

Soluble recombinant GFP was added to the bacterial suspension at 10 μg/ml 5 min prior to the experiments.

### Generation of a Ni-NTA functionalized glass surface for selective protein immobilization.

Addition of the Ni-NTA functionality to a glass surface was inspired by existing protocols (53, 54). Glass coverslips (#1.5) were placed in a holder and sonicated in acetone for 30 min. The coverslips were then rinsed with MilliQ water, dried with a stream of nitrogen gas, and plasma treated for 10 min at maximal power (Zepto; Diener Electronic). The plasma-treated coverslips were then transferred into 150 ml of 1% (vol/vol) (3-aminopropyl)triethoxysilane (APTES) (Sigma-Aldrich) in toluene (Sigma-Aldrich) and stirred for 30 min. The coverslips were then rinsed in 150 ml of toluene for 10 min, dried by a stream of nitrogen gas, and then baked at 80°C for 45 min. The coverslips were then cooled down with a stream of nitrogen gas and transferred into a 150-ml stirred solution of 2 mg/ml *p*-phenylene diisothiocyanate (PDITC) (Sigma-Aldrich) in 10% (vol/vol) anhydrous pyridine (Sigma-Aldrich) and 90% (vol/vol) *N*,*N*-dimethylformamide (DMF) (Sigma-Aldrich) for 2 h in darkness. The coverslips were then flushed with 1 volume of absolute ethanol, followed by a wash in acetone for 10 min and drying with a stream of nitrogen gas. Then, half the coverslips were laid on a flat surface. We then prepared a solution of 457 mM *N*,*N*-bis(carboxymethyl)-l-lysine-hydrate (Sigma-Aldrich) in 1 M NaHCO_3_ (Sigma-Aldrich). Ninety microliters of the *N*,*N*-bis(carboxymethyl)-l-lysine-hydrate solution was deposited onto the coverslips and then sandwiched with another coverslip on top. These were incubated overnight at room temperature. The unreacted PDITC was then blocked by immersing the coverslips into a solution of 5 mg/ml bovine serum albumin (BSA) plus 5% ethanolamine in phosphate-buffered saline (PBS) for 30 min. The slides were then washed in 1× PBS for 10 min under constant stirring, transferred into a solution of 1% (wt/vol) solution of nickel sulfate (NiSO_4_) for 1 h under stirring, and then washed in 1× PBS for 10 min followed by a second wash in 0.1× PBS for 10 min and dried under a stream of nitrogen gas. Fifty microliters of recombinant GFP at 1 mg/ml was deposited onto each coverslip and incubated over 2 days in the dark at 4°C. The slides were again flushed in 1× PBS for 10 min followed by a second wash in 0.1× PBS for 10 min and then dried with a stream of nitrogen.

### Visualization.

For widefield visualizations, we used a Nikon TiE epifluorescence microscope equipped with a Hamamatsu Orca Flash 4 camera and an oil immersion 100× Plan Apo numerical aperture (NA) 1.45 objective.

For all time-lapses and mammalian cell visualizations, we used a Nikon Eclipse Ti2-E inverted microscope coupled with a Yokogawa CSU W2 confocal spinning disk unit and equipped with a Prime 95B scientific complementary metal oxide semiconductor (sCMOS) camera (Photometrics). For time-lapses, we used a 40× objective with an NA of 1.15 to acquire z-stacks with 2-μm intervals over 6 μm. Each plane was acquired at low laser power for 200 ms, allowing us to threshold out free bacteria in flow from bound bacteria. For stained mammalian cell visualizations, we used a 100× oil immersion objective with an NA of 1.45 to acquire z-stacks with 0.5-μm intervals.

We used NIS Elements (Nikon) for three-dimensional rendering of z-stack pictures.

### Flow experiments and data acquisition.

Bacteria induced overnight were diluted 1:10 in Fluorobrite-10% FBS-1% GlutaMAX and loaded in syringes. We applied equivalent mean flow rates according to the different channel dimensions in Fig. 2. Shear stress at the centerline was calculated using the formula: shear stress = 6 × flow × kinematic viscosity/(channel width × channel height^2^) (55). Flow generating shear stress of 0.05 Pa at the channel centerline (unless stated otherwise) was applied using syringe pumps connected to microchannels seeded with induced HeLa cells at 50 to 80% confluence or to channels functionalized with GFP. z-stacks for bacterial contact efficiency were generated by confocal microscopy every second. Three different fields of view were sequentially imaged for 5 min per biological replicate.

Data to model residence time were generated by confocal microscopy of z-stacks every 10 s. Three different fields of view were simultaneously imaged for 60 min per biological replicate. Cell surface area was acquired once in the green channel at the start of the experiment. Number of HeLa cells was then approximated based on their average size as manually determined with 5 biological replicates of 3 frames each.

Illustrative confocal time-lapse with both channels for GFP and mScarlet was acquired at either 2 or 6 stacks per min at ×100 magnification.

### Bacterium tracking.

We use the maximum-intensity projection of full stacks to detect attaching bacteria. We used the Fiji plugin Trackmate with LoG detector (56). Threshold was set so that >95% of bacteria are detected on the final frame and <5% of the tracks were false positive (two different bacteria slowing down in the same area on consecutive frames). The LAP tracker was used with 5-μm maximal interframe distance and gap closing, track splitting, and closing with a maximal distance of 3 μm. Final number of spots and tracks and spot statistics were exported for data analysis.

### Data analysis and modeling.

Data generated by Trackmate were analyzed using Matlab. In brief, contact efficiency was defined as the number of tracks strictly longer than 2 frames, divided by the total number of contacts (bacterium appearing on one frame or more). Bacteria present from the first frame were removed from the analysis to exclude bacteria that attached during handling time.

Residence times of tracks strictly longer than two frames were considered and sorted in a histogram of 10-s bins. We further transformed these data into an “inverse” cumulative histogram to present results in a manner classical for adhesion events by defining:

fraction remaining (t=20  s)=  total number of tracks on three fields of view

fraction remaining (t + 10) =  fraction remaining (t) − number of tracks of duration t

Because many bacteria were bound at the end of the acquisition, we had to circumvent the artificial stop of tracks. To do so, we considered the binding events occurring within the first 30 min and followed them over 30 additional minutes for the fitting. We fitted the fraction remaining as a function of residence time with a dual exponential decay as follows:

$$\text{fraction remaining }\left(t\right)=C_{\text{transient}}\times e^{-\;\frac{t}{{Τ}_{\text{transient}}}}\;+\;C_{\text{res}}\times e^{-\;\frac{t}{{Τ}_{\text{res}}}}$$

The raw data for all experiments are summarized in Table S2 in the supplemental material.

### Static coculture and mammalian cell staining.

Mammalian cells were coincubated with bacteria for 5 h 30 min at a multiplicity of infection (MOI) of 50 (Fig. 1C) or for 1 h at an MOI of 200 (Fig. 3A and B). Wells were washed once with PBS, fixed in 4% paraformaldehyde for 20 min, permeabilized with 0.1% Triton X-100 for 5 min, and washed twice with PBS. Phalloidin-Atto 655 (Sigma) was used to stain actin at 500 nM for 15 min. 4′,6-Diamidino-2-phenylindole (DAPI) was used for nuclear counterstain at 1 μM for 5 min. Cells were washed twice with PBS and imaged by confocal microscope at ×100 magnification.

### Bacterial staining, titration, and quantification.

Bacteria displaying VHH were washed with PBS and stained with recombinant GFP at 100 μg/ml for 10 min prior to two PBS washes and imaging under a 1% agarose-PBS pad. Widefield fluorescent pictures were taken at ×100 and ×1.5 lens magnification.

### Production of recombinant proteins.

eGFP sequence (GenBank accession no. 8382257) was cloned into pET28a (Novagen, https://www.merckmillipore.com/CH/de/product/pET-28a+-DNA-Novagen,EMD_BIO-69864) in frame with an N-terminal 6×His tag, and the resulting pXP226 was retransformed into the BL21 strain. Production was induced with 1 mM IPTG (isopropyl-β-d-thiogalactopyranoside; Fisher Bioreagents) at 20°C overnight. Bacteria were pelleted and lysed by sonication in lysis buffer (Tris 100 mM, NaCl 0.5 M, glycerol 5%), and eGFP was purified using fast-flow His-affinity columns (GE Healthcare) and eluted with 500 mM imidazole. Buffer was exchanged to PBS using 30-kDa ultracentrifugation spin columns (Merck), and aliquots at 1 mg/ml were snap-frozen for further use. mKate2 was produced using the same protocol using the plasmid SpyTag003-mKate2 (Addgene 133452).

### Additional materials.

Movies S1 to S6 and their corresponding legends can be found on Zenodo (https://doi.org/10.5281/zenodo.5079719).

10.1128/mBio.01392-21.1 TEXT S1 Supplementary methods and references. Download Text S1, DOCX file, 0.02 MB. Copyright © 2021 Pierrat et al. 2021 Pierrat et al. https://creativecommons.org/licenses/by/4.0/ This content is distributed under the terms of the Creative Commons Attribution 4.0 International license.

## References

[1] Nadell CD, Drescher K, Foster KR. 2016. Spatial structure, cooperation and competition in biofilms. Nat Rev Microbiol 14:589–600. doi:10.1038/nrmicro.2016.84. 27452230

[2] Pizarro-Cerdá J, Cossart P. 2006. Bacterial adhesion and entry into host cells. Cell 124:715–727. doi:10.1016/j.cell.2006.02.012. 16497583

[3] Viela F, Mathelié-Guinlet M, Viljoen A, Dufrêne YF. 2020. What makes bacterial pathogens so sticky? Mol Microbiol 113:683–690. doi:10.1111/mmi.14448. 31916325

[4] Petrova OE, Sauer K. 2012. Sticky situations: key components that control bacterial surface attachment. J Bacteriol 194:2413–2425. doi:10.1128/JB.00003-12. 22389478 PMC3347170

[5] Berne C, Ellison CK, Ducret A, Brun YV. 2018. Bacterial adhesion at the single-cell level. Nat Rev Microbiol 16:616–627. doi:10.1038/s41579-018-0057-5. 30008468

[6] Thomas WE, Trintchina E, Forero M, Vogel V, Sokurenko EV. 2002. Bacterial adhesion to target cells enhanced by shear force. Cell 109:913–923. doi:10.1016/S0092-8674(02)00796-1. 12110187

[7] Kolewe KW, Zhu J, Mako NR, Nonnenmann SS, Schiffman JD. 2018. Bacterial adhesion is affected by the thickness and stiffness of poly(ethylene glycol) hydrogels. ACS Appl Mater Interfaces 10:2275–2281. doi:10.1021/acsami.7b12145. 29283244 PMC5785418

[8] Lecuyer S, Rusconi R, Shen Y, Forsyth A, Vlamakis H, Kolter R, Stone HA. 2011. Shear stress increases the residence time of adhesion of *Pseudomonas aeruginosa*. Biophys J 100:341–350. doi:10.1016/j.bpj.2010.11.078. 21244830 PMC3021681

[9] de la Serna JB, Schütz GJ, Eggeling C, Cebecauer M. 2016. There is no simple model of the plasma membrane organization. Front Cell Dev Biol 4:106–117. doi:10.3389/fcell.2016.00106. 27747212 PMC5040727

[10] Ribet D, Cossart P. 2015. How bacterial pathogens colonize their hosts and invade deeper tissues. Microbes Infect 17:173–183. doi:10.1016/j.micinf.2015.01.004. 25637951

[11] Girard V, Mourez M. 2006. Adhesion mediated by autotransporters of Gram-negative bacteria: structural and functional features. Res Microbiol 157:407–416. doi:10.1016/j.resmic.2006.02.001. 16725315

[12] Nicolay T, Vanderleyden J, Spaepen S. 2015. Autotransporter-based cell surface display in Gram-negative bacteria. Crit Rev Microbiol 41:109–123. doi:10.3109/1040841X.2013.804032. 23855358

[13] Leo JC, Oberhettinger P, Schütz M, Linke D. 2015. The inverse autotransporter family: intimin, invasin and related proteins. Int J Med Microbiol 305:276–282. doi:10.1016/j.ijmm.2014.12.011. 25596886

[14] Kenny B, DeVinney R, Stein M, Reinscheid DJ, Frey EA, Finlay BB. 1997. Enteropathogenic *E. coli* (EPEC) transfers its receptor for intimate adherence into mammalian cells. Cell 91:511–520. doi:10.1016/S0092-8674(00)80437-7. 9390560

[15] Isberg RR, Leong JM. 1990. Multiple β1 chain integrins are receptors for invasin, a protein that promotes bacterial penetration into mammalian cells. Cell 60:861–871. doi:10.1016/0092-8674(90)90099-Z. 2311122

[16] Schulte R, Kerneis S, Klinke S, Bartels H, Preger S, Kraehenbuhl JP, Pringault E, Autenrieth IB. 2000. Translocation of *Yersinia enterocolitica* across reconstituted intestinal epithelial monolayers is triggered by *Yersinia* invasin binding to β1 integrins apically expressed on M-like cells. Cell Microbiol 2:173–185. doi:10.1046/j.1462-5822.2000.00047.x. 11207574

[17] Nägele V, Heesemann J, Schielke S, Jiménez-Soto LF, Kurzai O, Ackermann N. 2011. *Neisseria meningitidis* adhesin NadA targets β1 integrins: functional similarity to *Yersinia* invasin. J Biol Chem 286:20536–20546. doi:10.1074/jbc.M110.188326. 21471204 PMC3121457

[18] Ismaili A, Meddings JB, Ratnam S, Sherman PM. 1999. Modulation of host cell membrane fluidity: a novel mechanism for preventing bacterial adhesion. Am J Physiol Gastrointest Liver Physiol 277:201–208. 10.1152/ajpgi.1999.277.1.G201 10409168

[19] Dufrêne YF. 2015. Sticky microbes: forces in microbial cell adhesion. Trends Microbiol 23:376–382. doi:10.1016/j.tim.2015.01.011. 25684261

[20] Sauer MM, Jakob RP, Luber T, Canonica F, Navarra G, Ernst B, Unverzagt C, Maier T, Glockshuber R. 2019. Binding of the bacterial adhesin FimH to its natural, multivalent high-mannose type glycan targets. J Am Chem Soc 141:936–944. doi:10.1021/jacs.8b10736. 30543411

[21] Nilsson LM, Thomas WE, Trintchina E, Vogel V, Sokurenko EV. 2006. Catch bond-mediated adhesion without a shear threshold: trimannose versus monomannose interactions with the FimH adhesin of *Escherichia coli*. J Biol Chem 281:16656–16663. doi:10.1074/jbc.M511496200. 16624825

[22] Sauer MM, Jakob RP, Eras J, Badau S., Eriş D, Navarra G, Bernèche S, Ernst B, Maier T, Glockshuber R. 2016. Catch-bond mechanism of the bacterial adhesin FimH. Nat Commun 7:10738. doi:10.1038/ncomms10738. 26948702 PMC4786642

[23] Bell GI. 1978. Models for the specific adhesion of cells to cells. Science 200:618–627. doi:10.1126/science.347575. 347575

[24] Patel S, Mathivanan N, Goyal A. 2017. Bacterial adhesins, the pathogenic weapons to trick host defense arsenal. Biomed Pharmacother 93:763–771. doi:10.1016/j.biopha.2017.06.102. 28709130

[25] Misselwitz B, Kreibich SK, Rout S, Stecher B, Periaswamy B, Hardt W-D. 2011. *Salmonella enterica* serovar Typhimurium binds to HeLa cells via fim-mediated reversible adhesion and irreversible type three secretion system 1-mediated docking. Infect Immun 79:330–341. doi:10.1128/IAI.00581-10. 20974826 PMC3019867

[26] Salema V, Marín E, Martínez-Arteaga R, Ruano-Gallego D, Fraile S, Margolles Y, Teira X, Gutierrez C, Bodelón G, Fernández LÁ. 2013. Selection of single domain antibodies from immune libraries displayed on the surface of *E. coli* cells with two β-domains of opposite topologies. PLoS One 8:e75126. doi:10.1371/journal.pone.0075126. 24086454 PMC3781032

[27] Glass DS, Riedel-Kruse IH. 2018. A synthetic bacterial cell-cell adhesion toolbox for programming multicellular morphologies and patterns. Cell 174:649–658.e16. doi:10.1016/j.cell.2018.06.041. 30033369

[28] Weikum J, Kulakova A, Tesei G, Yoshimoto S, Jægerum LV, Schütz M, Hori K, Skepö M, Harris P, Leo JC, Morth JPP. 2020. The extracellular juncture domains in the intimin passenger adopt a constitutively extended conformation inducing restraints to its sphere of action. SSRN doi:10.2139/ssrn.3635798. PMC7718877 33277518

[29] Sabzevari H, Kantor J, Jaigirdar A, Tagaya Y, Naramura M, Hodge J, Bernon J, Schlom J. 2001. Acquisition of CD80 (B7-1) by T cells. J Immunol 166:2505–2513. doi:10.4049/jimmunol.166.4.2505. 11160311

[30] Kubala MH, Kovtun O, Alexandrov K, Collins BM. 2010. Structural and thermodynamic analysis of the GFP:GFP-nanobody complex. Protein Sci 19:2389–2401. doi:10.1002/pro.519. 20945358 PMC3009406

[31] Fridy PC, Li Y, Keegan S, Thompson MK, Nudelman I, Scheid JF, Oeffinger M, Nussenzweig MC, Fenyö D, Chait BT, Rout MP. 2014. A robust pipeline for rapid production of versatile nanobody repertoires. Nat Methods 11:1253–1260. doi:10.1038/nmeth.3170. 25362362 PMC4272012

[32] Ricci V, Galmiche A, Doye A, Necchi V, Solcia E, Boquet P. 2000. High cell sensitivity to *Helicobacter pylori* VacA toxin depends on a GPI-anchored protein and is not blocked by inhibition of the clathrin-mediated pathway of endocytosis. Mol Biol Cell 11:3897–3909. doi:10.1091/mbc.11.11.3897. 11071915 PMC15045

[33] Goddette DW, Frieden C. 1986. Actin polymerization. The mechanism of action of cytochalasin D. J Biol Chem 261:15974–15980. doi:10.1016/S0021-9258(18)66662-1. 3023337

[34] Nishida-Aoki N, Tominaga N, Kosaka N, Ochiya T. 2020. Altered biodistribution of deglycosylated extracellular vesicles through enhanced cellular uptake. J Extracell Vesicles 9:1713527. doi:10.1080/20013078.2020.1713527. 32082512 PMC7006786

[35] Howlader MA, Li C, Zou C, Chakraberty R, Ebesoh N, Cairo CW. 2019. Neuraminidase-3 is a negative regulator of LFA-1 adhesion. Front Chem 7:791. doi:10.3389/fchem.2019.00791. 31824923 PMC6882948

[36] Cruz-Chu ER, Malafeev A, Pajarskas T, Pivkin IV, Koumoutsakos P. 2014. Structure and response to flow of the glycocalyx layer. Biophys J 106:232–243. doi:10.1016/j.bpj.2013.09.060. 24411255 PMC3907246

[37] Dufrêne YF, Persat A. 2020. Mechanomicrobiology: how bacteria sense and respond to forces. Nat Rev Microbiol 18:227–240. doi:10.1038/s41579-019-0314-2. 31959911

[38] Tripathi P, Beaussart A, Alsteens D, Dupres V, Claes I, von Ossowski I, de Vos WM, Palva A, Lebeer S, Vanderleyden J, Dufrêne YF. 2013. Adhesion and nanomechanics of pili from the probiotic *Lactobacillus rhamnosus* GG. ACS Nano 7:3685–3697. doi:10.1021/nn400705u. 23531039

[39] McKenzie M, Ha SM, Rammohan A, Radhakrishnan R, Ramakrishnan N. 2018. Multivalent binding of a ligand-coated particle: role of shape, size, and ligand heterogeneity. Biophys J 114:1830–1846. doi:10.1016/j.bpj.2018.03.007. 29694862 PMC5937168

[40] Spaulding CN, Klein RD, Ruer S, Kau AL, Schreiber HL, Cusumano ZT, Dodson KW, Pinkner JS, Fremont DH, Janetka JW, Remaut H, Gordon JI, Hultgren SJ. 2017. Selective depletion of uropathogenic *E. coli* from the gut by a FimH antagonist. Nature 546:528–532. doi:10.1038/nature22972. 28614296 PMC5654549

[41] Tollis S, Dart AE, Tzircotis G, Endres RG. 2010. The zipper mechanism in phagocytosis: energetic requirements and variability in phagocytic cup shape. BMC Syst Biol 4:149. doi:10.1186/1752-0509-4-149. 21059234 PMC2991294

[42] Romero S, Le Clainche C, Gautreau AM. 2020. Actin polymerization downstream of integrins: signaling pathways and mechanotransduction. Biochem J 477:1–21. doi:10.1042/BCJ20170719. 31913455

[43] González C, Chames P, Kerfelec B, Baty D, Robert P, Limozin L. 2019. Nanobody-CD16 catch bond reveals NK cell mechanosensitivity. Biophys J 116:1516–1526. doi:10.1016/j.bpj.2019.03.012. 30979550 PMC6486492

[44] Yakovenko O, Nunez J, Bensing B, Yu H, Mount J, Zeng J, Hawkins J, Chen X, Sullam PM, Thomas W. 2018. Serine-rich repeat adhesins mediate shear-enhanced streptococcal binding to platelets. Infect Immun 86:e00160-18. doi:10.1128/IAI.00160-18. 29581195 PMC5964516

[45] Klamecka K, Severin PM, Milles LF, Gaub HE, Leonhardt H. 2015. Energy profile of nanobody-GFP complex under force. Phys Biol 12:056009. doi:10.1088/1478-3975/12/5/056009. 26356046

[46] Delanoë-Ayari H, Al Kurdi R, Vallade M, Gulino-Debrac D, Riveline D. 2004. Membrane and acto-myosin tension promote clustering of adhesion proteins. Proc Natl Acad Sci U S A 101:2229–2234. doi:10.1073/pnas.0304297101. 14982992 PMC356933

[47] Arabyan N, Park D, Foutouhi S, Weis AM, Huang BC, Williams CC, Desai P, Shah J, Jeannotte R, Kong N, Lebrilla CB, Weimer BC. 2016. *Salmonella* degrades the host glycocalyx leading to altered infection and glycan remodeling. Sci Rep 6:29525. doi:10.1038/srep29525. 27389966 PMC4937416

[48] Johansson MM, Bélurier E, Papageorgiou AC, Sundin AP, Rahkila J, Kallonen T, Nilsson UJ, Maatsola S, Nyholm TKM, Käpylä J, Corander J, Leino R, Finne J, Teneberg S, Haataja S. 2020. The binding mechanism of the virulence factor *Streptococcus suis* adhesin P subtype to globotetraosylceramide is associated with systemic disease. J Biol Chem 295:14305–14324. doi:10.1074/jbc.RA120.014818. 32796033 PMC7573278

[49] Mühlenkamp M, Oberhettinger P, Leo JC, Linke D, Schütz MS. 2015. *Yersinia* adhesin A (YadA) - beauty & beast. Int J Med Microbiol 305:252–258. doi:10.1016/j.ijmm.2014.12.008. 25604505

[50] Krachler AM, Orth K. 2013. Targeting the bacteria-host interface strategies in anti-adhesion therapy. Virulence 4:284–294. doi:10.4161/viru.24606. 23799663 PMC3710331

[51] McKenzie GJ, Craig NL. 2006. Fast, easy and efficient: site-specific insertion of transgenes into enterobacterial chromosomes using Tn 7 without need for selection of the insertion event. BMC Microbiol 6:39. doi:10.1186/1471-2180-6-39. 16646962 PMC1475584

[52] Datsenko KA, Wanner BL. 2000. One-step inactivation of chromosomal genes in *Escherichia coli* K-12 using PCR products. Proc Natl Acad Sci U S A 97:6640–6645. doi:10.1073/pnas.120163297. 10829079 PMC18686

[53] Chevalier S, Cuestas-Ayllon C, Grazu V, Luna M, Feracci H, de la Fuente JM. 2010. Creating biomimetic surfaces through covalent and oriented binding of proteins. Langmuir 26:14707–14715. doi:10.1021/la103086b. 20795718

[54] Bañuls MJ, Puchades R, Maquieira Á. 2013. Chemical surface modifications for the development of silicon-based label-free integrated optical (IO) biosensors: a review. Anal Chim Acta 777:1–16. doi:10.1016/j.aca.2013.01.025. 23622959

[55] Persat A, Nadell CD, Kim MK, Ingremeau F, Siryaporn A, Drescher K, Wingreen NS, Bassler BL, Gitai Z, Stone HA. 2015. The mechanical world of bacteria. Cell 161:988–997. doi:10.1016/j.cell.2015.05.005. 26000479 PMC4451180

[56] Tinevez J-Y, Perry N, Schindelin J, Hoopes GM, Reynolds GD, Laplantine E, Bednarek SY, Shorte SL, Eliceiri KW. 2017. TrackMate: an open and extensible platform for single-particle tracking. Methods 115:80–90. doi:10.1016/j.ymeth.2016.09.016. 27713081

