# Supplementary figures and images for: The Mammalian Membrane Microenvironment Regulates the Sequential Attachment of Bacteria to Host Cells
Source: mBio. 2021 Aug 3;12(4):e01392-21. doi: 10.1128/mBio.01392-21 (PMC8406306; doi:10.1128/mBio.01392-21)

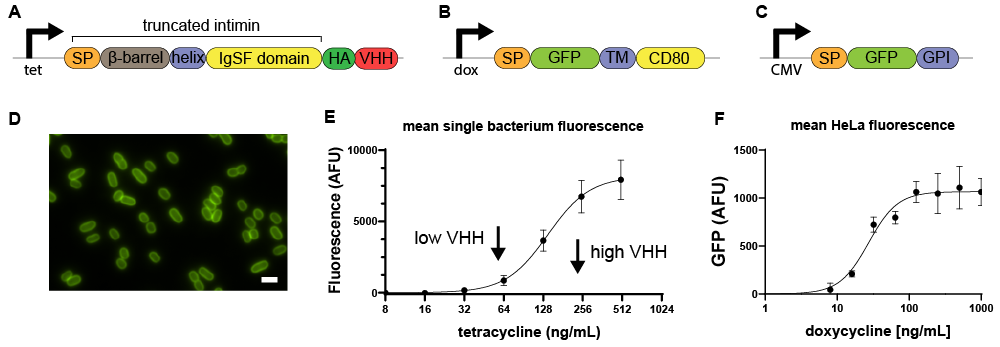

Supplement: FIG S1 [file mbio.01392-21-sf001.tif]

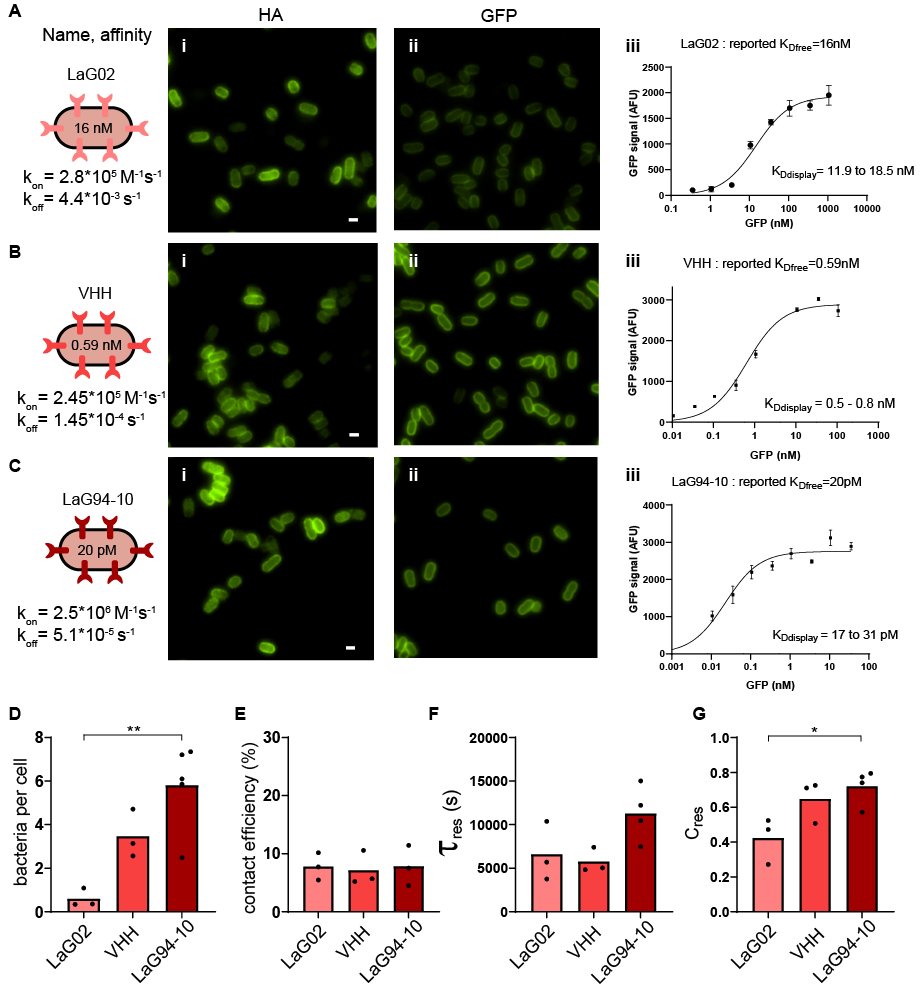

Supplement: FIG S2 [file mbio.01392-21-sf002.tif]

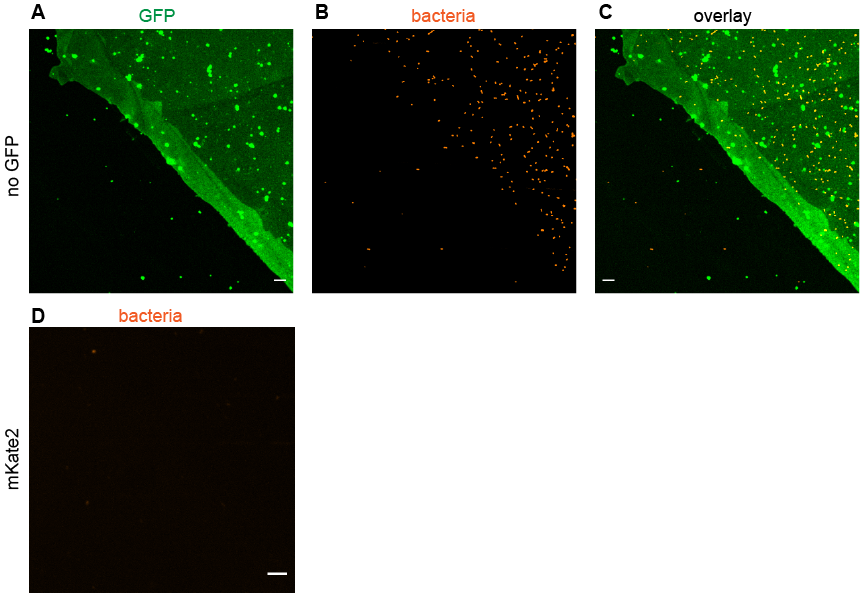

Supplement: FIG S3 [file mbio.01392-21-sf003.tif]

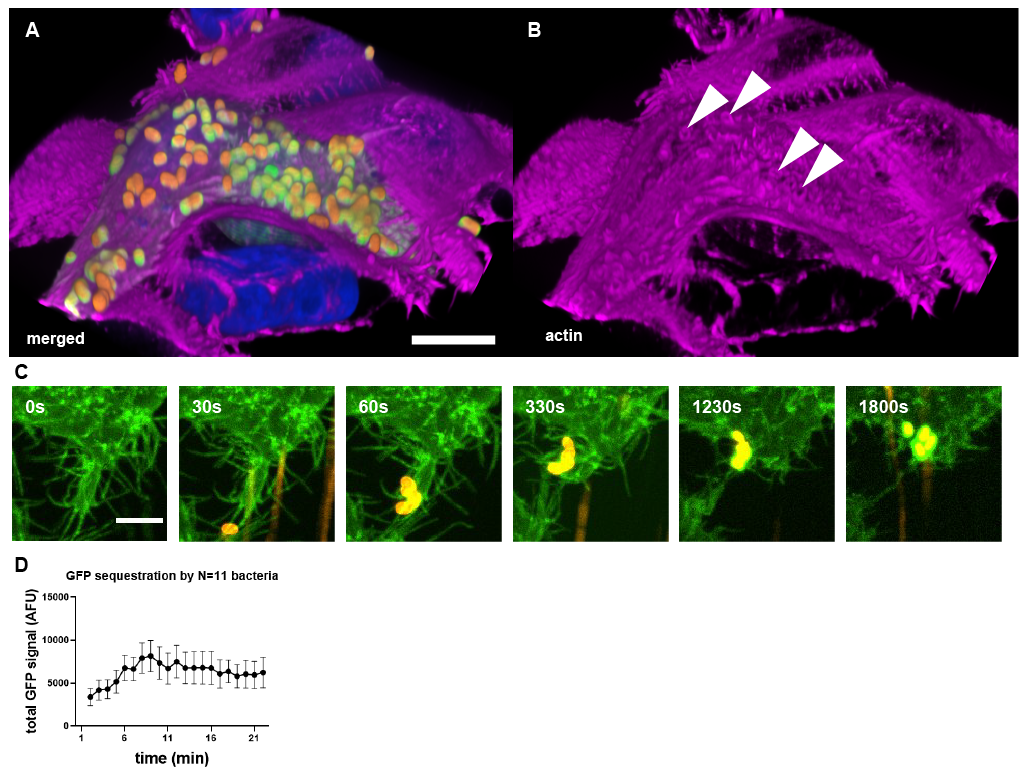

Supplement: FIG S4 [file mbio.01392-21-sf004.tif]

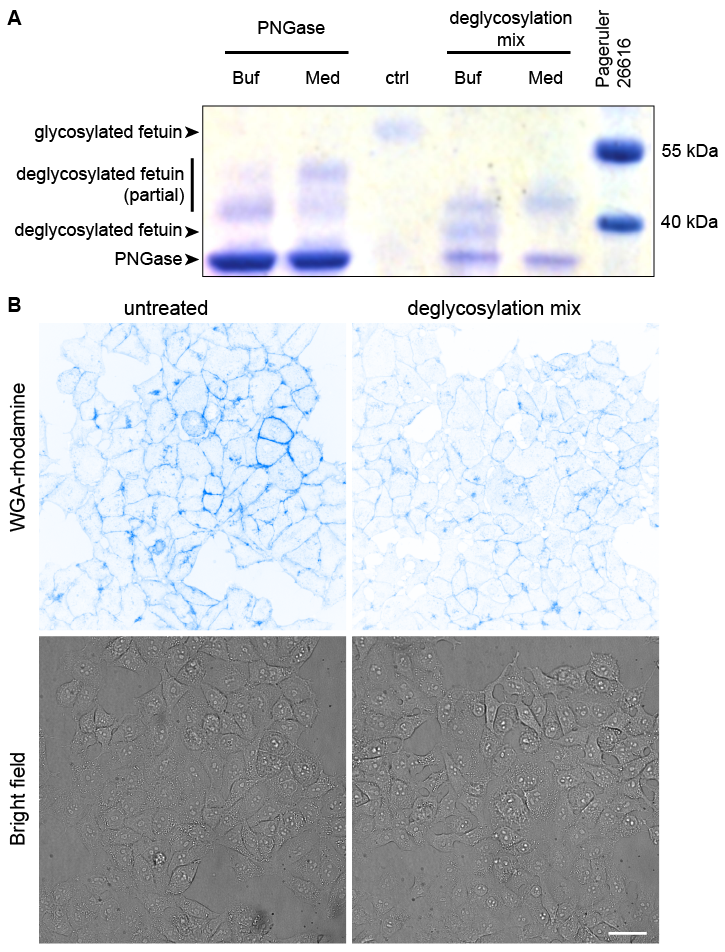

Supplement: FIG S5 [file mbio.01392-21-sf005.tif]

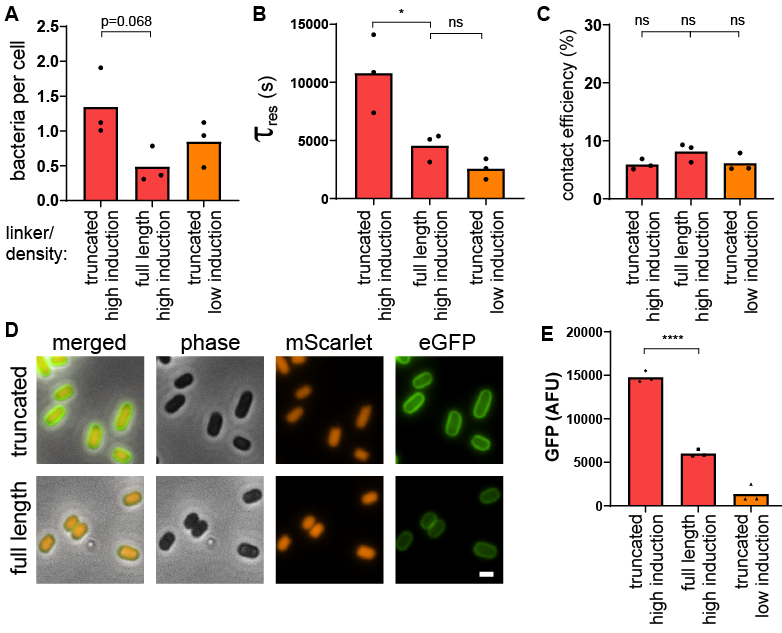

Supplement: FIG S6 [file mbio.01392-21-sf006.tif]
