# Supplementary material for: The Mammalian Membrane Microenvironment Regulates the Sequential Attachment of Bacteria to Host Cells
Source: mBio. 2021 Aug 3;12(4):e01392-21. doi: 10.1128/mBio.01392-21 (PMC8406306; doi:10.1128/mBio.01392-21)
Supplement: TABLE S1 [file mbio.01392-21-st001.docx]

**Supplementary table 1: Plasmid cloning strategy and primers**

| Plasmid | Description, cloning | Sources |
| --- | --- | --- |
| pDSG323 | Empty display control, Kan^R^  Tet-inducible truncated intimin construct | Glass et al ^1^, Addgene 115594 |
| pDSG339 | VHH anti GFP display. Kan^R^ , K_D_ = 0.59 nM  Tet-inducible VHH display based on truncated intimin | Glass et al^1^ |
| PeGFP_GPI | pEGFP-N1 - preproinsulinSP eGFP linker DAF GPI  eGFP display for mammalian cells , anchoring motif from CD55, preproinsulin secretion peptide | Ricci et al ^2^  Generous gift from Prof. Van der Goot, EPFL |
| pXP145 | Constituve eGFP (N105Y, E125V,Y146F) display based on C-terminal CD80 anchor, Amp^R^, Neo^R^.  Digestion and ligation of the following:  Backbone: pCDNA3* XbaI HindIII  Insert: pENTR_SignalPeptide-GFP(N105Y/E124V/Y145F-superfastFolding** mutation)_mCD80TransMembrane XbaI HindIII | *Invitrogen  **Generous gift form Prof. Joerg Huelsken, EPFL  This study |
| pXP226 | eGFP in pET28a for recombinant expression  Gibson assembly with the following PCR products:  pET28a* oXP546 oXP547  pUCBB** oXP333 oXP334 | *EMD Biosciences  **Vick et al^3^,  Addgene 32548  This study |
| pXP327 | Dox-inducible eGFP (N105Y, E125V,Y146F) display based on CD80 anchor on lentivector, Amp^R^, Neo^R^  Digestion and ligation of the following:  Backbone: pCW57-RFP-P2A-MCS* EcoRI BamHI  Insert: pXP145 first amplified with oXP799 oXP800 then EcoRI BamHI | *Barger et al^4^, Addgene 78933  This study |
| pXP340 | Dox-inducible eGFP (N105Y, E185V,Y206F) display based on CD80 anchor on lentivector, Amp^R^, Neo^R^  Digestion and ligation of the following:  Backbone: pRRLSIN.cPPT.GFP.WPRE* SacII NotI  Insert: pXP327 SacII NotI | *Trono lab, EPFL, unpublished  Addgene 12252  This study |
| pXP383 | VHH anti GFP display. Kan^R^ , K_D_ = 0.59 nM  Tet-inducible VHH display based on truncated intimin + HA tag.  Gibson assembly with the following PCR products:  pDSG339 oXP912 oXP926  pDSG339 oXP528 oXP913 | This study |
| pXP384 | Low affinity VHH anti GFP display. Kan^R^ , K_D_ = 16 nM  Tet-inducible VHH display based on truncated intimin + HA tag.  Gibson assembly with the following PCR products:  pDSG339* oXP914 oXP915  LaG02* oXP916 oXP917 | *Fridy et al ^5^  This study |
| pXP388 | High affinity VHH anti GFP display. Kan^R^ , K_D_ = 20 pM  Tet-inducible VHH display based on truncated intimin + HA tag.  Gibson assembly with the following PCR products:  pDSG339* oXP914 oXP915  LaG94-10* oXP924 oXP925 | *Fridy et al ^5^  This study |
| pZA002 | pGRG36 j23119_mScarlet  Constitutive synthetic promoter driving the expression of mScarlet in Tn7 vector.  Digestion and ligation of the following:  Backbone: pGRG36* PacI XhoI  Insert: synthetized j23119**_mScarlet PacI XhoI | *McKenzie et al ^6^  Addgene 16666  **parts.igem.org/  Part:BBa_J23119  This study |
| SpyTag003-mKate2 | Expresses SpyTag003-mKate2 (a far-red fluorescent protein) in bacterial cytoplasm | Keeble et al, ^7^  Addgene 133452 |
|  |  |  |
| Primers | Sequence |  |
| oXP333 | ATGGTGAGCAAGGGCGAG |  |
| oXP334 | TCACTTGTACAGCTCGTCC |  |
| oXP528 | ttaccaatgcttaatcagtgagg |  |
| oXP546 | ATGGACGAGCTGTACAAGTGAGATCCGGCTGCT |  |
| oXP547 | CTCGCCCTTGCTCACCATGCTGCTGTGATGATGATG |  |
| oXP799 | agcgaattcgccaccatggactcc |  |
| oXP800 | cccggatccctaaaggaagacggtctgttc |  |
| oXP851 | atcaggcaatttggcgttgccgtcagtctcagttaatcaggttacaacgagtgtaggctggagctgcttc |  |
| oXP852 | agaagcgtagccgtaatcggattattcgcgagccatcgactcattcagatggtccatatgaatatcctccttagttcc |  |
| oXP912 | TACCCGTATGATGTTCCCGACTATGCCatggctcaggtgcagctg |  |
| oXP913 | GGCATAGTCGGGAACATCATACGGGTAtctagtCGCACCATCAAAAAATATAAC |  |
| oXP914 | TAATAAtactagtagcggccgc |  |
| oXP915 | GGCATAGTCGGGAACATCATAC |  |
| oXP916 | GTTCCCGACTATGCCATGGCCCAAGTTCAGCT |  |
| oXP917 | cgctactagtaTTATTATACAGTAACCTGTGTTCCCTG |  |
| oXP924 | GTTCCCGACTATGCCATGGCTCAAGTCCAGCTTG |  |
| oXP925 | gctactagtaTTATTAACTGACGGTCACCTGC |  |
| oXP926 | gagtcaggcaactatggatgaac |  |
