# Supplementary material for: The Mammalian Membrane Microenvironment Regulates the Sequential Attachment of Bacteria to Host Cells
Source: mBio. 2021 Aug 3;12(4):e01392-21. doi: 10.1128/mBio.01392-21 (PMC8406306; doi:10.1128/mBio.01392-21)
Supplement: TEXT S1 [file mbio.01392-21-t0001.docx]

**Supplementary methods**

**K_D(display)_ measurements**

To measure VHH affinity when displayed at the bacterium surface, serial dilution of GFP were performed by increasing the volume to avoid antigen depletion. For each concentration, 100 µL of bacteria induced with 250 ng/mL tetracycline overnight were washed with PBS and stained for two hours. For volumes below 50 mL, bacteria were pelleted and resuspended in 4% PFA in PBS. For volumes above 50 mL, bacteria were retrieved on 0.22 µm filters using 4% PFA in PBS. Bacteria were then imaged under a 1% agarose PBS pad. Wide field fluorescent pictures were taken at 100x and 1.5x lens magnification.

Using Fiji software, bacteria were detected using the mScarlet channel and the corresponding regions of interests were used to quantify mean GFP intensity for each bacterium. Prism software (Graphpad) was used to perform a non-linear fit of the mean GFP signal among bacteria on the field of views using the formula “One site – specific binding” Y = max * [GFP] / (K_D_ + [GFP]) and estimate K_Ddisplay_.

**N-acetyl glucosamine staining of live HeLa cells**

HeLa cells were cultured in DMEM (Thermofisher) supplemented with 10% FBS (Life Technologies) at 37°C and 5% CO2. One microliter of Protein Deglycosylation Mix II (NEB) of was added per well of Ibidi 96-well plate for overnight treatment (150 µL total). Cell supernatant was replaced with 100 µL of 10 µg/mL rhodamine-labelled wheat germ agglutinin (Vectorlabs) in PBS for 30 min at room temperature. Supernatant was replaced by 200 µL PBS for confocal imaging.

**HA tag staining**

Bacteria harboring a HA tag were washed with PBS and stained with anti-HA antibody conjugated with FITC (Abcam ab1208) at 10 µg/mL for 75 minutes in the dark, washed once with PBS and imaged under a 1% agarose PBS pad. Widefield fluorescent pictures were taken at 100x and 1.5x lens magnification.

**GFP uptake rate**

*E. coli* VHH were added under static conditions at a MOI of 200 for a couple of minutes and washed 3 times before widefield epifluorescence imaging. HeLa GFP captured 11 bacteria. Image segmentation performed using the red channel (*E.coli*) to quantify the local total GFP signal around bacteria over time.

**SI references**

1. Glass, D. S. & Riedel-Kruse, I. H. A Synthetic Bacterial Cell-Cell Adhesion Toolbox for Programming Multicellular Morphologies and Patterns. *Cell* **174**, 649-658.e16 (2018).

2. Ricci, V. *et al.* High cell sensitivity to Helicobacter pylori VacA toxin depends on a GPI-anchored protein and is not blocked by inhibition of the clathrin-mediated pathway of endocytosis. *Mol. Biol. Cell* **11**, 3897–3909 (2000).

3. Vick, J. E. *et al.* Optimized compatible set of BioBrick^TM^ vectors for metabolic pathway engineering. *Appl. Microbiol. Biotechnol.* **92**, 1275–1286 (2011).

4. Barger, C. J., Branick, C., Chee, L. & Karpf, A. R. Pan-cancer analyses reveal genomic features of FOXM1 overexpression in cancer. *Cancers (Basel).* **11**, (2019).

5. Fridy, P. C. *et al.* A robust pipeline for rapid production of versatile nanobody repertoires. **11**, (2014).

6. Mckenzie, G. J. & Craig, N. L. Fast , easy and efficient : site-specific insertion of transgenes into Enterobacterial chromosomes using Tn 7 without need for selection of the insertion event. **7**, 1–7 (2006).

7. Keeble, A. H. *et al.* Approaching infinite affinity through engineering of peptide-protein interaction. *Proc. Natl. Acad. Sci. U. S. A.* **116**, 26523–26533 (2019).
